# Development Using Bioluminescence Imaging of a Recombinant Anguillid Herpesvirus 1 Vaccine Candidate Associated with Normal Replication In Vitro but Abortive Infection In Vivo

**DOI:** 10.3390/vaccines12121423

**Published:** 2024-12-17

**Authors:** Haiyan Zhang, Arun Sridhar, Natacha Delrez, Bo He, Sophie Fourny, Yuan Gao, Owen Donohoe, Alain F. C. Vanderplasschen

**Affiliations:** 1 Immunology-Vaccinology, Department of Infectious and Parasitic Diseases, Fundamental and Applied Research for Animals & Health (FARAH), Faculty of Veterinary Medicine, University of Liège, B-4000 Liège, Belgium; haiyanzhang199104@163.com (H.Z.); arun.sridhar@uliege.be (A.S.); natacha.delrez@ifremer.fr (N.D.); bo.he@doct.uliege.be (B.H.); sophie.fourny@uliege.be (S.F.); gaoyuan@bio-ss.net (Y.G.);; 2 Bioscience Research Institute, Technological University of the Shannon, N37 HD68 Athlone, Westmeath, Ireland; 3 WEL Research Institute, Avenue Pasteur 6, B-1300 Wavre, Belgium

**Keywords:** Anguillid herpesvirus 1, AngHV-1, Cyvirus anguillidallo 1, alloherpesvirus, cyprinivirus, Cyvirus, anguillid eel, European eel, recombinant vaccine, in vivo bioluminescent imaging

## Abstract

Background/Objectives: Anguillid herpesvirus 1 (AngHV-1) (recently renamed Cyvirus anguillidallo 1) is the etiologic agent of a lethal disease that affects several eel species. It is thought to be one of the main infectious agents causing a population decline in wild eels and economic loss within the eel aquaculture sector. To date, no vaccines are available against AngHV-1. Recently, we developed a safe and efficacious live attenuated recombinant vaccine against Cyprinid herpesvirus 3 (CyHV-3). This CyHV-3 recombinant vaccine encodes a deletion of ORF57. Orthologues of CyHV-3 ORF57 exist in Cyprinid herpesvirus 2 (CyHV-2, ORF57) and AngHV-1 (ORF35). Methods: In the present study, using recombinant strains and bioluminescent in vivo imaging, we investigated the effect of AngHV-1 ORF35 deletion on virus replication in vitro, virulence in vivo, and the potential of an AngHV-1 ORF35-deleted recombinant as a vaccine candidate for the mass vaccination of eels by immersion. With this goal in mind, we produced ORF35-deleted recombinants using two parental strains: a UK strain and a recombinant derived from the former strain by insertion of a Luciferase–GFP reporter cassette into a non-coding intergenic region. Results: Analyses of ORF35-deleted recombinants led to the following observations: (i) AngHV-1 ORF35 is not essential for viral growth in cell culture, and its deletion does not affect the production of extracellular virions despite reducing the size of viral plaque. (ii) In contrast to what has been observed for CyHV-3 ORF57 and CyHV-2 ORF57, in vivo bioluminescent analyses revealed that AngHV-1 ORF35 is an essential virulence factor and that its deletion led to abortive infection in vivo. (iii) Inoculation of the AngHV-1 ORF35-deleted recombinant by immersion induced a protective immune response against a wild-type challenge. This protection was shown to be dose-dependent and to rely on the infectivity of AngHV-1 ORF35-deleted virions. Conclusions: This study suggests that the AngHV-1 ORF35 protein has singular properties compared to its orthologues encoded by CyHV-2 and CyHV-3. It also supports the potential of AngHV-1 ORF35-deleted recombinants for the mass vaccination of eels by immersion.

## 1. Introduction

The genus *Cyprinivirus* (recently renamed *Cyvirus*) belongs to the family *Alloherpesviridae* of the order *Herpesvirales.* This genus comprises alloherpesviruses of cyprinids (*Cyprinid herpesvirus 1*, *2*, and *3* (CyHV-1, -2, and -3), recently renamed *Cyvirus cyprinidallo 1*, *2*, and *3*)) and anguillid eels (*Anguillid herpesvirus 1* (AngHV-1) recently renamed *Cyvirus anguillidallo 1*). CyHV-1 and CyHV-3 infect common and koi carp *(Cyprinus carpio* species); CyHV-2 infects goldfish, Crucian carp, and Gibel carp (all of which are *Carassius* species); and AngHV-1 infects different anguillid eel species (*Anguilla*). Notably, unlike cyprinids, anguillid eels are catadromous fish [1]. Their lifecycle begins in the open ocean, with leptocephali larvae drifting thousands of kilometers across oceans to reach continental coastal waters, where they metamorphose into glass eels. These juvenile eels then migrate upstream towards freshwater habitats, where they grow into yellow eels over several years. Finally, they mature into silver eels and migrate back to the spawning site where they initially hatched to spawn before dying.

Since the first report of AngHV-1 in the 1980s, it has been detected globally, affecting different anguillid eel species from temperate to tropical countries [2,3]. It is causing economical losses in eel aquaculture [4,5] and is having a negative impact on wildlife. Despite their isolation in different eel species and from a broad geographic origin, AngHV-1 isolates were found to have a low genetic diversity [6].

Infection with AngHV-1 causes severe hemorrhagic skin and gill lesions and an abnormal swimming posture [7]. AngHV-1 disease is associated with high morbidity and with a mortality rate varying between eel species, for instance, 30% in American eels (*Anguilla rostrata*) [8] and up to 100% for short-finned eels (*Anguilla bicolor*) [5]. In addition to its negative impact on the aquaculture sector, AngHV-1 has also been suggested as an important cause of the decline in the wildlife of anguillid eel species such as European eels (*Anguilla anguilla*) [9,10]. The numbers of glass eels reaching European coasts now amount to only 1% of those estimated in the early 1980s. As a result of disease outbreaks and several other factors, European eels have been classified as critically endangered on the International Union for Conservation of Nature (IUCN) Red List of Threatened Species [11,12]. The potential impact of AngHV-1 on wild European eels together with its negative impact on this species in the aquaculture sector (with a mortality rate of up to 30% [9]) stimulated the development of an efficacious vaccine compatible with mass vaccination.

To date, there is no vaccine against AngHV-1 available on the market [9]. Current measures to prevent AngHV-1 disease within aquaculture settings mainly rely, when possible, on shifting water temperatures outside of the range (below 22 °C) associated with efficient lytic replication and clinical disease [13]. This approach acts as an effective way to reduce the clinical symptoms of infected eels and can reduce economic losses. However, it promotes latent infection [14], thereby increasing the risk of transmission when latently infected eels are latter cohabiting with naïve eels under stressing conditions [15]. Thus, fish originating from such facilities, including those used in restocking programs, could be a potential source for AngHV-1’s spread to wildlife [16]. Only a few studies address the development of AngHV-1 candidate vaccines. Lately, a formalin-inactivated AngHV-1 vaccine tested in American eels (*Anguilla rostrata*) was reported effective in inducing high levels of specific antibody and protection against a challenge [17]. However, this inactivated vaccine requires two intramuscular injections per fish. It is therefore incompatible with the mass vaccination of eels and with the vaccination of relatively small subjects. The development of a safe and efficacious attenuated recombinant vaccine could circumvent these limitations. This was the main goal of the present study.

Recently, we developed an attenuated recombinant vaccine against CyHV-3 using prokaryotic recombination technologies and in vivo bioluminescent imaging system (IVIS) [18]. The development of this vaccine relied on the deletion of CyHV-3 ORF56 and ORF57 [18]. This candidate vaccine was shown to exhibit interesting properties such as, efficient replication in a cell culture (even if reduced compared to the parental wild-type strain), a good safety profile in juvenile carp, and the ability to induce protective immunity at the portal of entry. Further studies demonstrated that the deletion of CyHV-3 ORF57 was sufficient to induce this safety-efficacy profile [19].

Orthologues of CyHV-3 ORF57 exist in CyHV-1 and CyHV-2 (ORF57) and in AngHV-1 (ORF 35) [19,20]. The roles of these orthologues are still unknown. The effect of ORF57 deletion on CyHV-2 infection has been tested in vitro and in vivo using a recombinant strain in which ORF57 was replaced by an expression cassette encoding a transgenic antigen [21]. Surprisingly, and in contrast to what was observed for CyHV-3, ORF57 deletion did not impair CyHV-2 replication in a cell culture and induced only a mild reduction of virulence in vivo. Together, these results suggest that CyHV-3 ORF57 orthologues encoded by cypriniviruses express different biological roles despite their phylogenetic relationship.

Here, we studied the effect of ORF35 deletion on the ability of AngHV-1 to replicate in cell culture and on its virulence after inoculation of yellow eels by different routes. These experiments demonstrated that ORF35 deletion does not affect the production of AngHV-1 virions in cell culture but led to an abortive infection in vivo. Next, we studied the potential of an AngHV-1 ORF35-deleted recombinant as a vaccine candidate for the mass vaccination of yellow eels by immersion in water containing the virus. Exposure to the AngHV-1 ORF35-deleted recombinant induced a protective immune response against a wild-type challenge. This protection was shown to be dose-dependent and to rely on the infectivity of the AngHV-1 ORF35-deleted recombinant. This study suggests that the AngHV-1 ORF35 protein has singular properties compared to its orthologues encoded by CyHV-2 and CyHV-3. It also supports the potential of AngHV-1 ORF35-deleted recombinants for the mass vaccination of eels by immersion.

## 2. Materials and Methods

### 2.1. Cells and AngHV-1 Strains

**Figure 1:**
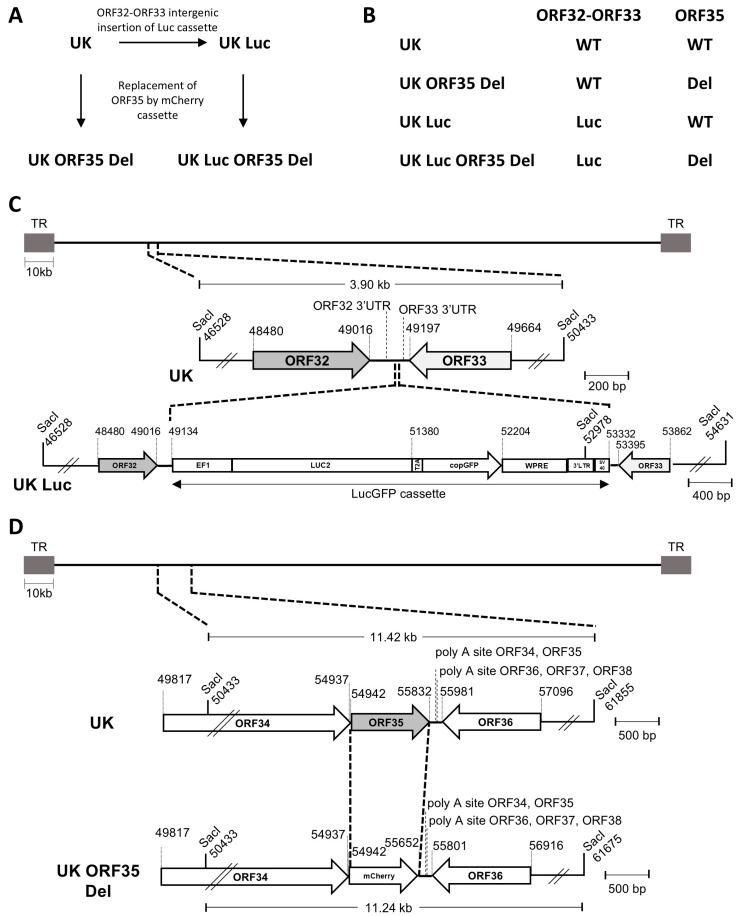
Schematic representation of the strategy used to produce AngHV-1 recombinants by homologous directed recombination (HDR). (**A**) Flowchart of the production of the UK Luc and ORF35 Del recombinant strains by HDR in eukaryotic cells. (**B**) Genotype of the UK parental strain and derived recombinant strains for the ORF32–ORF33 intergenic region and the ORF35 locus. WT, wild-type; Luc, inserted LucGFP cassette; Del, deleted. (**C**) A schematic representation of the genome structure of UK Luc. The genome of AngHV-1 flanked by two terminal repeats (LTR and RTR) and the intergenic ORF32–ORF33 genome region are shown at the top. (**D**) Schematic representation of the genome structure of the UK ORF35 Del recombinant. The genome of AngHV-1 flanked by two terminal repeats (LTR and RTR) and the ORF35 genome region are shown at the top. In panels (**C**,**D**), SacI restriction sites and predicted restriction fragments (in kb) are shown. Coordinates are those of the AngHV-1 reference strain available in GenBank (accession number: MW580855.1).

Eel kidney (EK-1) cells were cultured as described previously [6]. The AngHV-1 isolate (hereafter called the UK strain) used in this study was kindly provided by Dr Keith Way (Centre for Environment, Fisheries, and Aquaculture Science, United Kingdom). The recombinant AngHV-1 UK Luc strain was produced by homologous directed recombination (HDR) using the UK strain as a parental strain in a recent study (Delrez et al., manuscript submitted for publication). The UK Luc strain encodes a LucGFP cassette inserted in the intergenic region ORF32–ORF33 of the AngHV-1 genome between the coordinates 49,134 and 49,135 (MW580855.1) (Figure 1). The LucGFP cassette consists of an EF1 promoter driving the transcription of a bicistronic mRNA-encoding firefly luciferase 2 (LUC2) and copepod GFP (copGFP) proteins linked by a T2A peptide (Figure 1C).

### 2.2. Production of Recombinant Strains of AngHV-1 Deleted for ORF35 by HDR in Eukaryotic Cells

The UK ORF35 Del and UK Luc ORF35 Del strains were produced by HDR in EK-1 cells using the UK and the UK Luc strains as parental strains, respectively (Figure 1). The pGEMT mCherry vector containing the recombination fragment consisted of the mCherry ORF flanked by 500 bp sequences, corresponding to the end of AngHV-1 ORF34 for the left homology region and the ORF35–ORF36 intergenic region plus the end of ORF36 for the right homology region. One day after transfection with the pGEMT mCherry vector, EK-1 cells were infected with AngHV-1 UK or UK Luc strains at a multiplicity of infection (MOI) of 0.01 plaque forming unit (pfu)/cell. Four days later, the supernatant was collected and diluted to infect naive EK-1 cells. Infected cells were overlaid with a medium containing carboxymethylcellulose (CMC) [6]. Viral plaques expressing mCherry were picked and amplified until we obtained 100% plaque-expressing mCherry.

### 2.3. Genetic Characterization of AngHV-1 Recombinants

The molecular structures of all recombinant strains were confirmed by monitoring SacI restriction fragment length polymorphism by agarose gel electrophoresis, PCR of the recombination loci, and full-length genome sequencing, as described previously [6].

### 2.4. Transcriptional Analyses

EK-1 cells were mock-infected or infected at an MOI of 0.1 pfu/cell. Twenty-four hours post infection (pi), RNA was isolated using the NucleoSpin RNA Mini kit (Macherey-Nagel, Düren, Germany), and residual DNA was removed using the TURBO DNA-free Kit (Invitrogen, Waltham, MA, USA). Reverse transcriptase (RT) reactions were performed on 5 µg of RNA using Superscript III Reverse Transcriptase and with oligo(dT) primers (Invitrogen) to generate cDNA. ORF32, ORF33, ORF34, ORF35, ORF36, and ORF55 (AngHV-1 DNA polymerase, based on Rijsewijk et al. [22]) were amplified using the pairs of primers listed in Table S1 using Phusion High-Fidelity DNA Polymerase (New England Biolabs, Ipswich, MA, USA). To exclude amplification from contaminant viral genomic DNA in purified RNA, PCR reactions were performed when RT was omitted from the reactions.

### 2.5. Indirect Immunofluorescence Staining

Cells were fixed in phosphate-buffered saline (PBS) containing 4% (*w*/*v*) paraformaldehyde (PAF) at 4 °C for 15 min and then 20 °C for 15 min. After washing with PBS, samples were permeabilized in PBS containing 0.1% (*v*/*v*) NP-40 at 37 °C for 15 min. Immunofluorescent staining (incubation and washes) was performed in PBS containing 10% fetal calf serum (FCS) (*v*/*v*) (PBS–FCS). Rabbit polyclonal antibodies (pAbs) raised against AngHV-1-purified virions (diluted 1:2000), following a procedure similar to He et al. [23], were used as the primary antibody. The primary antibody was incubated at 37 °C for 1 h. After washing with PBS–FCS, Alexa Fluor 488 goat anti-rabbit immunoglobulin G (H+L) (Invitrogen) was used as the secondary antibody (diluted 1:1000) in PBS–FCS. The secondary antibody was incubated at 37 °C for 30 min. After washing with PBS–FCS, cells were incubated with PBS containing DAPI (dilute 1:2000) (ThermoFisher, Waltham, MA, USA) at room temperature for 5 min then washed with PBS before mounting.

### 2.6. Viral Growth Assay

Triplicate cultures of EK-1 cells were infected with AngHV-1 at an MOI of 0.01 pfu/cell. After an incubation period of 2 h, the cells were washed with PBS and overlaid with culture medium. The cell supernatant was collected at successive intervals. After centrifugation at 900× *g* for 30 min at 4 °C to pellet cell debris, the supernatant was collected and stored at −80 °C. Viral titration was carried out by triplicate plaque assays in EK-1 cells, as described previously [6].

### 2.7. Viral-Plaque-Area Assay

Viral-plaque-area assays were performed, as described previously [6]. Individual plaques were revealed by immunofluorescent staining and imaged using a Nikon A1R confocal microscope, and areas were measured using the ImageJ software [24].

### 2.8. Viral Photoinactivation by Psoralen/UV Treatment

To inactivate the UK ORF35 Del recombinant strain without affecting viral structural proteins (and, consequently, the ability of the virus to enter cells susceptible to AngHV-1 infection), virions were submitted to a treatment associating incubation with psoralen (4′-Aminomethyltrioxsalen hydrochloride, Sigma-Aldrich, St. Louis, MO, USA) and exposure to long-wave UV light (365 nm, 6 W, 0.16 A, UVP UVL-56, Analytik Jena, USA). In brief, 1 × 10^5^ pfu of UK ORF35 Del were incubated in 1 mL of L-15 medium containing 5 µg/mL of psoralen and incubated on ice in a 35 mm sterile plastic Petri dish under UV light for 10 min at a distance of 5 cm. The efficiency of viral inactivation by psoralen/UV treatment was controlled by titration of residual infectious viral particles on EK-1 cells.

### 2.9. Fish

European eels at the glass eel developmental stage were obtained from an accredited commercial company (Foucher Maury, Paimboeuf, France). Microbiological, parasitic, and clinical examinations were conducted immediately after arrival in the animal facility and then on a monthly basis to control fish health. Glass eels were grown in 40 L freshwater tanks maintained at 25 °C. Fish were used at the stage of yellow eel.

### 2.10. AngHV-1 Inoculation Modes

Different modes of inoculation were used in this study. (i) Inoculation by intraperitoneal (IP) injection: Eels were anesthetized by immersion in water containing benzocaine (25 mg/L of water). A volume of 20 µL of a culture medium containing 2 × 10^5^ pfu of AngHV-1 was injected intraperitoneally using a 0.3 mL insulin syringe (BD Micro-Fine). After injection, fish were placed in a recovery bath, then returned to their tanks. (ii) Inoculation by immersion in infectious water: fish were inoculated by immersion in water containing AngHV-1 (doses used (pfu/mL) are described in the figure legends) for 2 h under constant aeration. (iii) Inoculation by intradermic infection: Eels were anesthetized by immersion in water containing benzocaine (25 mg/L of water). Intradermal injections were administered using an electronic tattooing device. As part of this process, 20 µL of a culture medium containing 2 × 10^5^ pfu of AngHV-1 was added into a sterile tattoo needle ink reservoir. Using this device, inoculum was delivered intradermally at three sites (all on the left side of the body, including the front, middle, and end) in a linear pattern along a length of approximately 1 cm per site. After intradermal injections, the fish were placed in a recovery bath, then returned to their tanks.

### 2.11. Ethical Statement

The experiments, maintenance, and care of the fish complied with the guidelines of the European Convention for the Protection of Vertebrate Animals used for experimental and other Scientific Purposes (CETS n° 123). The animal studies were approved by the local ethics committee of the University of Liège, Belgium (laboratory accreditation no., 1610008; protocol no., 1896). All efforts were made to apply the “3Rs rules” to reduce the number of subjects used to minimize suffering and to improve fish welfare. Of note, the present study respected strict ending-points, implying that fish were euthanized before natural death. As a consequence, the present study did not rely on the read-out of the mortality rate.

### 2.12. Bioluminescent Imaging

Firefly (*Photinus pyralis*) luciferase activity was imaged using an IVIS (IVIS spectrum, PerkinElmer, Waltham, MA, USA), as described previously [18,25]. For cell culture analyses, the culture medium was replaced with a fresh medium containing D-luciferin (150 μg/mL) (Caliper LifeSciences, Hopkinton, MA, USA). Analyses were performed after an incubation period of 10 min at room temperature. For in vivo analyses, fish were anesthetized with benzocaine (25 mg/L of water). Fifteen minutes before a bioluminescence analysis, D-luciferin (150 mg/kg of body weight) was injected into the peritoneal cavity. The fish were analyzed in vivo lying on their right and left sides and ex vivo after euthanasia with benzocaine (250 mg/L of water). Dissected organs were analyzed independently from the body. All images were acquired using a maximum auto-exposure time of 1 min, a binning factor of 8, and a f/stop of 1. The relative intensities of transmitted light from bioluminescence were determined automatically and represented as a pseudo-color image ranging from violet (least intense) to red (most intense) using the Living Image 4.7.3 software. Regions of interest (ROIs) were drawn manually by tracing the organs or body outlines, and the average radiance (p/sec/cm^2^/sr) was taken as the final measure of the bioluminescence emitted over the ROI. For the skin, the average radiance was measured on both sides of the body, and the results for individual fish were expressed as the mean of both sides. For the gills (mean of left and right gills) and internal organs, the analysis was performed ex vivo and separately. The cut-off for positivity was based on the mean + 3 SD of the values obtained for mock-infected fish (not represented in figures). For cell monolayers, the entire well was used as the ROI, and photon flux was measured in photons/sec (p/sec).

### 2.13. Statistical Analyses

**Figure 2:**
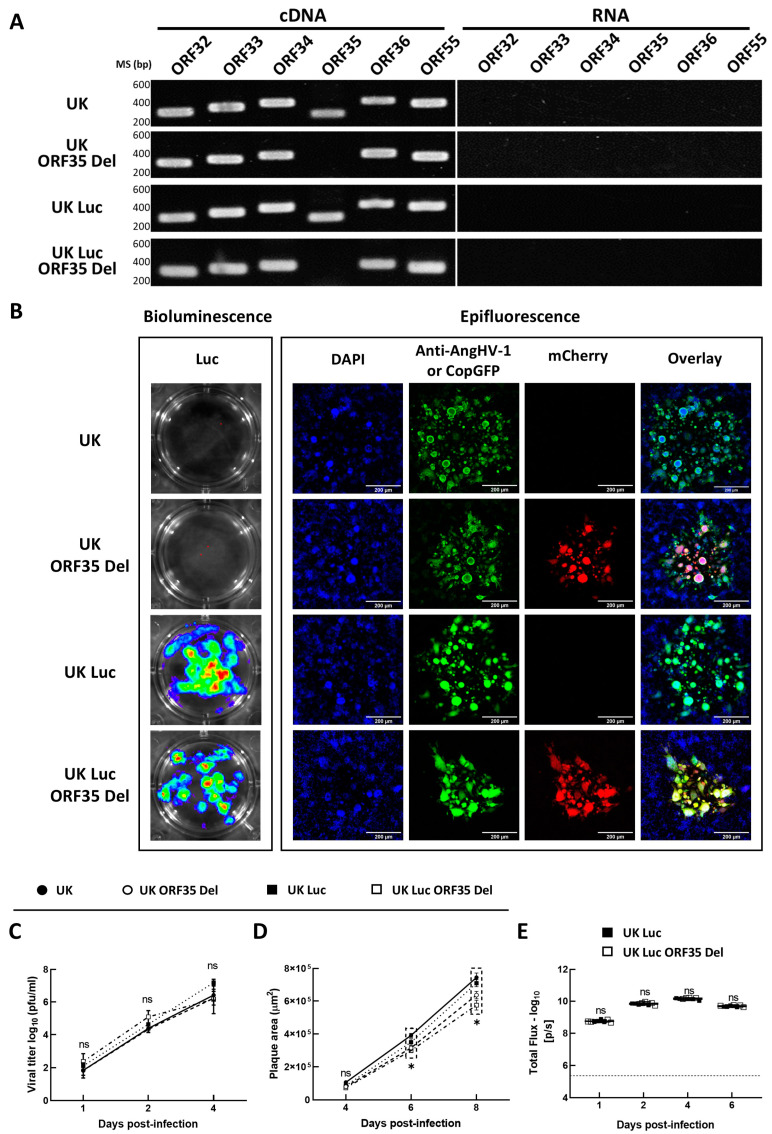
Characterization of AngHV-1 recombinant strains. (**A**) Transcriptional analysis of genes ORF32, ORF33, ORF34, ORF35, ORF36, and ORF55 expressed by the indicated strains of AngHV-1. ORF55 (AngHV-1 DNA polymerase) expression was used as a control. Marker sizes (MSs) in base pairs (bps) are indicated on the left. The left part and the right part of the figure represent the results of the PCR performed on cDNA and RNA, respectively. (**B**) Expression of reporter genes. EK-1 cells grown in 12-well plates were infected with the indicated strains, then overlaid with a medium containing CMC. At 4 dpi, infected cells were analyzed for the expression of bioluminescence and epifluorescence. The Luc signal was detected using the IVIS system (left frame). The reporters (copGFP and mCherry) and immunofluorescent staining (anti-AngHV-1) were detected by epifluorescent microscopy. Plaques of UK and UK ORF35 Del were revealed by indirect immunofluorescent staining (anti-AngHV-1) (right frame). (**C**) The replication kinetics and (**D**) viral plaque sizes of the ORF35-deleted strains were compared with those of the parental UK and UK Luc strains. (**E**) Luc expression of AngHV-1 recombinant strains (UK Luc and UK Luc ORF35 Del). The replication kinetic data represent the mean ± SEM of triplicate measurements. The data on the plaque area are the mean ± SEM of twenty measurements. The data on Luc expression are the mean ± SEM of triplicate measurements. The horizontal dashed line in panel **E** shows the mean + 3 SD of the data obtained for control non-infected cultures. Results of statistical comparisons between ORF35 Del strains and parental strains are indicated as follows: ns, no significant differences; *, *p* < 0.05.

**Figure 3:**
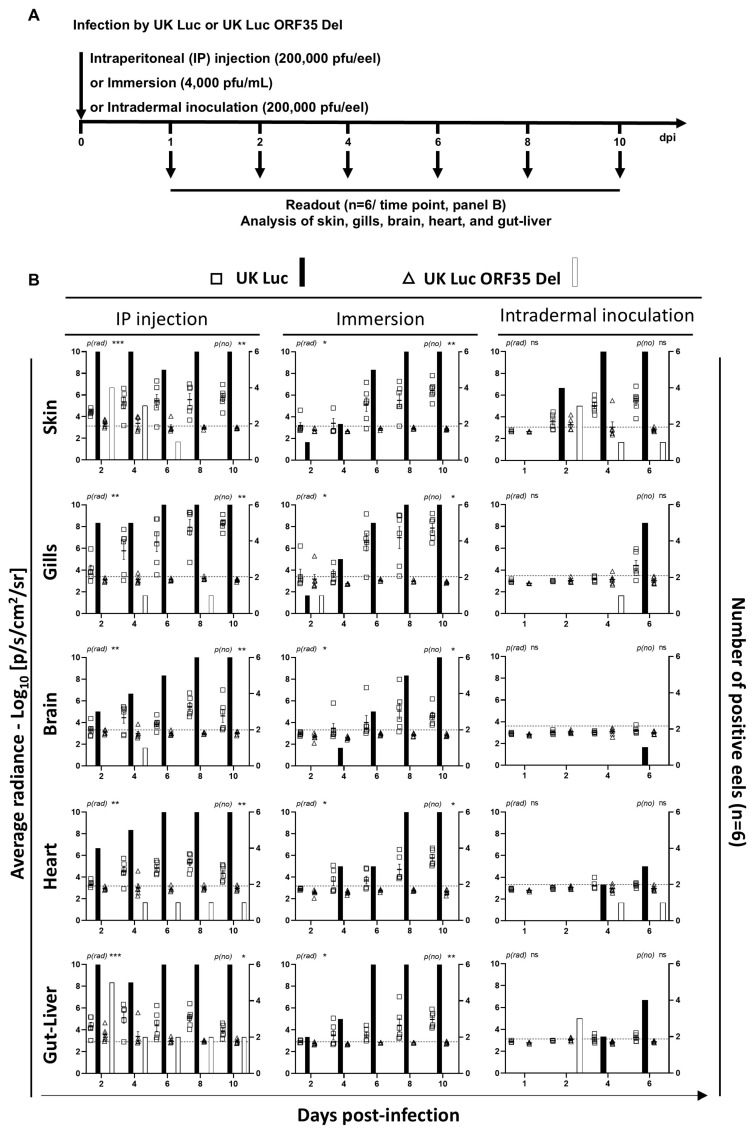

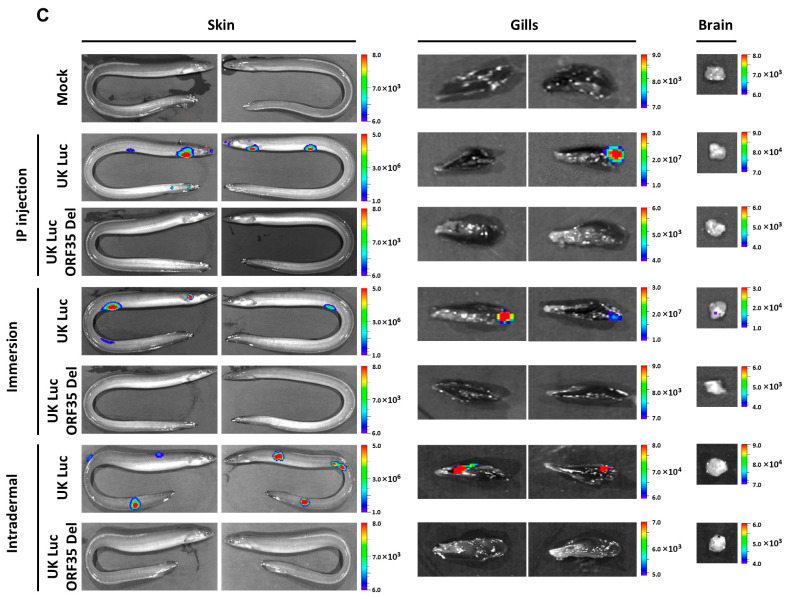
Effect of ORF35 deletion on AngHV-1 replication in vivo. (**A**) Flowchart of the experiment. At the time of inoculation, yellow eels (12.06 ± 2.72 g, mean ± SD) were mock-infected or infected with the indicated strains using different routes: IP injection of 200,000 pfu/eel or immersion in water containing 4000 pfu/mL or intradermal inoculation of 200,000 pfu/eel. At the indicated times post-infection, eels (*n* = 6, consisting of two eels from triplicate tanks) were imaged using an IVIS. (**B**) The effects of AngHV-1 infection routes: IP injection, immersion, and intradermal inoculation are presented in the left column, middle column, and right column, respectively. Average radiance (individual values, mean ± SEM) measured on the entire body surface of the fish, i.e., skin (individual values represent the mean values obtained for the left and right sides of each fish), gills (individual values represent the mean values obtained for the left and right gills), brain, heart, and gut-liver, were analyzed by IVIS (*n* = 6 per timepoint). The dashed line represents the threshold of positivity, which is the mean + 3 SD of the values obtained for the mock-infected fish (data not presented). The number of positive fish among the six analyzed fish is represented by bars (right axis). The average emitted radiances (*p*(*rad*)) of UK Luc were compared with UK Luc ORF35 Del using unpaired *t*-test (two-tail, Gaussian distribution) or Wilcoxon test (non-Gaussian distribution). The number of positive fish per group was compared between two strains using the Fisher–Pitman permutation test (*p*(*no*)). *p* values are represented as follows: ns, not significant; *, *p* < 0.05; **, *p* < 0.01; ***, *p* < 0.001. (**C**) Representative images of IVIS data (skin, gills, and brain) are presented. Eels with the closest scores to the mean of each infection route were selected for image illustration.

**Figure 4:**
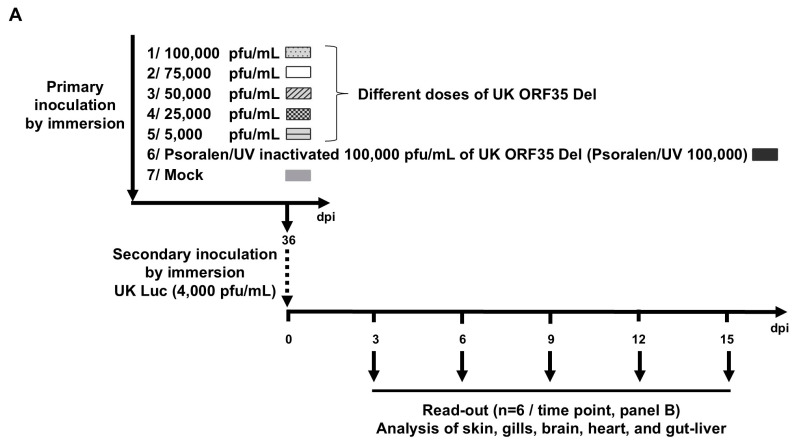

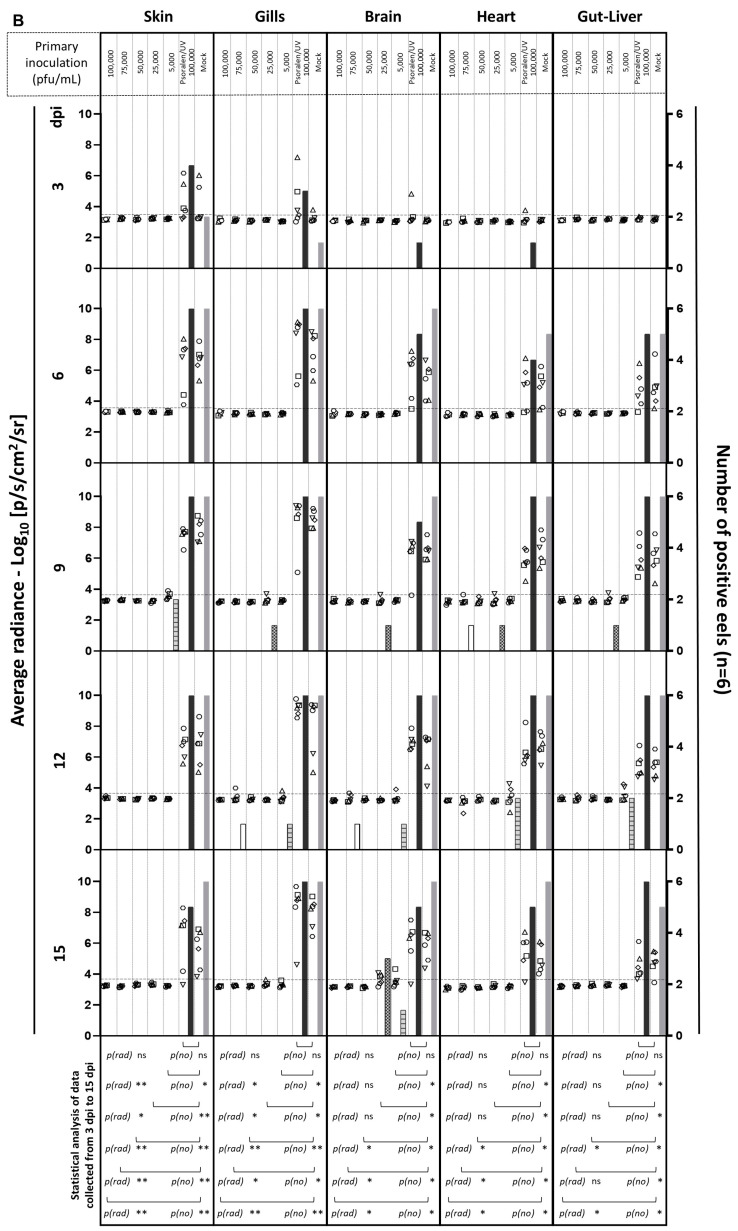

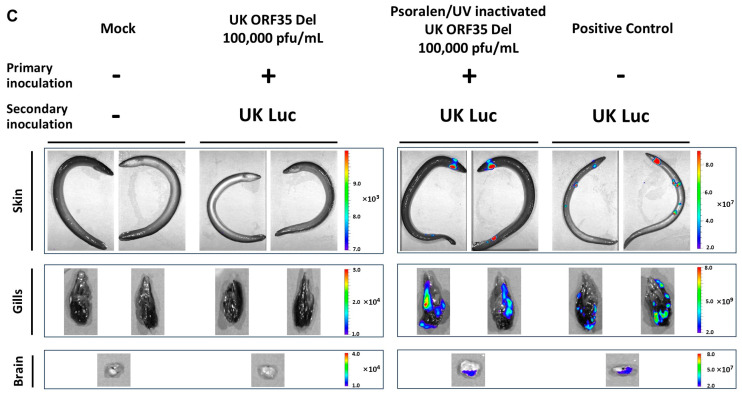
Dose-protection effect conferred by UK ORF35 Del in vivo. (**A**) Flowchart of the experiment. At the time of primary inoculation, yellow eels (28.52 ± 7.30 g, mean ± SD) were mock-infected or infected for 2 h by immersion in water containing the indicated doses of UK ORF35 Del or 100,000 pfu/mL of UK ORF35 Del strain treated by psoralen/UV to inactivate virus infectivity. At 36 days post-primary inoculation, eels were infected for 2 h by immersion in water containing 4000 pfu/mL of UK Luc expressing luciferase as a reporter. At the indicated times post-secondary inoculation, eels (*n* = 6, consisting of two eels from triplicate tanks) were imaged using IVIS. (**B**) Average radiance (individual values, mean ± SEM) measured on the entire body surface of fish, i.e., skin (individual values represent the mean values obtained for the left and right sides of each fish), gills (individual values represent the mean values obtained for the left and right gills), brain, heart, and gut–liver, were analyzed by IVIS (*n* = 6 per timepoint). The average radiance (*p*(*rad*)) of each group was compared with the “primary mock-infected group” using a non-parametric Kruskal–Wallis test followed by multiple comparisons with the two-stage step-up method of Benjamini, Krieger, and Yekutieli. Throughout this panel, the data obtained for every individual eel (within each group) are represented by the same symbol to allow for a correlation of the data obtained for the different organs at a specific dpi. The dashed line represents the threshold of positivity, which was calculated by the mean + 3 SD of the values obtained for the mock fish. The number of positive fish among the six analyzed fish is represented by bars (right axis). The positive fish (*p*(*no*)) from each group was compared with the primary mock-infected group using the Fisher–Pitman permutation test. *p* values are represented as follows: ns, not significant; *, *p* < 0.05; **, *p* < 0.01. (**C**) Representative images of IVIS data (skin, gills, and brain) are presented in the lower panel. Eels with the closest scores to the mean of each group (mock; UK ORF35 Del, 100,000 pfu/mL; psoralen/UV-inactivated UK ORF35 Del, 100,000 pfu/mL; and mock-infected with UK Luc) were selected for image illustration.

Residuals for each dataset were first tested for normality using the Shapiro–Wilk test (GraphPad Prism v8.0.1). Omnibus tests on data on viral growth, plaque size, and bioluminescence (Figure 2C–E) were conducted using either two-way ANOVA (GraphPad Prism v8.0.1) for datasets exhibiting a Gaussian distribution or Durbin tests for datasets exhibiting a non-Gaussian distribution (with the PMCMRplus package (v1.9.6) [26] in R (v4.2.2) [27]). Post-hoc multiple comparisons between groups of interest were made using either Sidak tests (two groups) or pairwise Tukey tests (more than two groups) (Graphpad Prism v8.0.1) for data exhibiting a Gaussian distribution and for data exhibiting a non-Gaussian distribution (using the wilcox.test function, a part of the R core package). The variables used for each omnibus test and their significance are described in the figure legends. The IVIS measurements in Figure 3 were compared using an unpaired *t*-test (two-tail) or a Wilcoxon test, as appropriate. The number of positive fish per group was compared between strains using Fisher–Pitman permutation test (using the coin package (v1.4.3) [28] in R). For the IVIS measurements in Figure 4, a non-parametric Kruskal–Wallis test was adopted, followed by controlling the False Discovery Rate (FDR) using the two-stage step-up method of Benjamini, Krieger, and Yekutieli (Graphpad Prism v8.0.1). The positive number of fish from each group was compared with a primary mock-infected group using the Fisher–Pitman permutation test. Results are indicated in each corresponding figure, with the statistical significance of all test results represented using the following symbols: *, *p* < 0.05; ** *p* < 0.01; *** *p* < 0.001.

## 3. Results

### 3.1. Production and Characterisation of AngHV-1 ORF35-Deleted Recombinant Strains

Recently, we demonstrated, using recombinant strains and bioluminescent imaging, that deletion of the ORF57 of CyHV-3 induces a reduction in viral growth in a cell culture and an attenuated phenotype in vivo [18,19]. Despite its attenuated phenotype, the CyHV-3 ORF57-deleted recombinant was shown to recapitulate the biological cycle of the wild-type parental strain but with a slower dissemination of the infection in the fish body and at a lower level and transient period of replication of the virus [18]. In the present study, we aimed to determine if the deletion of the orthologue of CyHV-3 ORF57 encoded by AngHV-1 (ORF35) also leads to such a phenotype in a cell culture and in vivo. With this goal in mind, different AngHV-1 recombinants were produced by HDR in eukaryotic cells (Figure 1A,B).

Two parental AngHV-1 strains were used for the production of AngHV-1 ORF35-deleted recombinants: the UK strain and the UK Luc recombinant strain derived from the former (Figure 1). The AngHV-1 UK strain was selected as a parental strain based on its efficient replication in cell culture and its ability to induce AngHV-1 disease after the inoculation of yellow eels by immersion in water containing the virus (Delrez et al., manuscript submitted for publication). As part of this earlier study, the UK Luc recombinant was derived from the UK strain by insertion of the LucGFP cassette in the non-coding ORF32–ORF33 intergenic region to allow the study of AngHV-1 in vivo by IVIS. The present study relied on this cutting-edge technique.

The ORF35-deleted recombinants were produced by HDR using both the UK and UK Luc strains as parental strains (Figure 1A,B). ORF35-deleted recombinants were produced by replacing ORF35 with a sequence encoding mCherry (Figure 1D) to allow for the identification of recombinant viral plaques under a fluorescence microscope. This approach generated the UK ORF35 Del and the UK Luc ORF35 Del recombinants. The molecular structure of these recombinants was controlled by PCR, restriction profile analyses, and full-length genome sequencing. The ability to produce the AngHV-1 ORF35-deleted strains using this approach demonstrated that ORF35 is not essential for viral replication in cell culture.

Next, to ensure that the mutation produced in the ORF35 locus has no effect on the expression of flanking genes (a potential polar effect), we compared the transcription of ORF34 and ORF36 in EK-1 cells after infection with the ORF35-deleted recombinants and their respective parental strains (Figure 2A). RT-PCR analyses demonstrated that the insertion had no major effect on the expression of flanking genes (Figure 2A, cDNA). The absence of contaminating viral genome in the purified RNA samples was controlled by running the PCR reactions on samples omitting the reverse transcription step (Figure 2A, RNA). No PCR products were detected. Similarly, a comparison of the transcription of ORF32 and ORF33 confirmed that the insertion of the LucGFP cassette has no significant impact on the expression of these genes (Figure 2A).

To confirm the expression of the expected reporter proteins by the recombinants, the two ORF35-deleted strains and their respective parental strains were analyzed for the expression of luciferase bioluminescence, and mCherry and copGFP fluorescences (Figure 2B). The results obtained confirm the expected pattern of reporter expression. The UK Luc and the UK Luc ORF35 Del strains expressed both luciferase bioluminescence (Figure 2B, left frame) and copGFP fluorescence (Figure 2B, right frame), while mCherry fluorescence correlated with ORF35 deletion (Figure 2B, right frame).

### 3.2. ORF35 Deletion Does Not Impair Production of AngHV-1 Virions in Cell Culture

As mentioned above, earlier studies demonstrated that the deletion of the orthologue of AngHV-1 ORF35 in CyHV-3 (ORF57) induced a reduction in virion production in a cell culture [18,19]. Here, we analyzed whether the deletion of AngHV-1 ORF35 also leads to a reduction in virion production in a cell culture. The replication kinetics of ORF35-deleted recombinant strains (UK ORF35 Del and UK Luc ORF35 Del strains) were compared with those of the parental strains (UK and UK Luc strains), as described in the materials and methods. Interestingly, all the recombinant strains reached high and comparable titers during the entire course of this experiment, with no significant statistical differences detected between the different strains (Figure 2C). The absence of a negative effect of ORF35 deletion on AngHV-1 viral replication in a cell culture was further supported by a comparison of the UK Luc ORF35 Del strain and the UK Luc strain measuring bioluminescence according to the time post infection (Figure 2E). No significant statistical difference was detected between the two strains at any of the timepoints post-inoculation. Together, these results demonstrate that deletion of ORF35 does not impair the production of AngHV-1 virions in cell culture. This result is in contrast to what was previously observed in CyHV-3 when deleting the orthologue of AngHV-1 ORF35 [18,19].

Next, we also investigated the effect of ORF35 deletion on the size of AngHV-1 plaques in cell culture. Plaque-area assays revealed that the two ORF35 Del strains produced significantly smaller plaques than the wild-type parental strains (two-way ANOVA, *p* < 0.0001 at 6 and 8 dpi). The post-hoc test revealed that the wild-type UK strain had a bigger plaque size than the UK ORF35 Del (*p* = 0.0120) and UK Luc ORF35 Del (*p* = 0.0013) strains at 6 dpi. At 8 dpi, post-hoc tests revealed that the UK strain had significantly larger plaques than both ORF35 Del recombinant strains: UK ORF35 Del (*p* < 0.0001) and UK Luc ORF35 Del (*p* < 0.0001). Notably, there was no difference between UK versus UK Luc and UK ORF35 Del versus UK Luc ORF35 Del in terms of viral titer or plaque size at any timepoint post-infection (Figure 2C,D).

Together, the results above demonstrate that ORF35 is not essential for AngHV-1 replication in a cell culture and that its deletion does not impair virion production despite slightly reducing the viral plaque size.

### 3.3. Effects of AngHV-1 ORF35 on Viral Replication In Vivo

The results above demonstrate that the UK versus UK Luc strains and the UK ORF35 Del versus UK Luc ORF35 Del strains exhibited comparable phonotypes in a cell culture. Consequently, we took advantage of the UK Luc and the UK Luc ORF35 Del strains to investigate the impact of ORF35 deletion on the AngHV-1 biological cycle in vivo using bioluminescence imaging.

Yellow eels were inoculated using three different inoculation methods: IP, immersion in water containing the virus, and intradermal inoculation (Figure 3A). For IP inoculation, each yellow eel was injected intraperitoneally with 2 × 10^5^ pfu and then analyzed by IVIS at 2, 4, 6, 8, and 10 dpi (Figure 3B, left column). The infection culminated in around 8 dpi in all organs tested. A comparison of the average radiance from both sides of the body, gills, brain, heart, and gut–liver tissues (taking each organ separately) revealed that in all organs, there were significant differences between the two strains, with the UK Luc ORF35 Del strain expressing either much lower or no bioluminescence signals. A comparison of the number of positive subjects also revealed significant differences between strains. The signal intensity and the number of positive subjects from the UK Luc strain remained higher than those of the UK Luc ORF35 Del strain up to the end of the experiment. The evolution of the signal observed for the UK Luc ORF35 Del strain suggested that the inoculation led to an erratic and abortive infection despite the artificial systemic mode of infection used.

Inoculation by immersion allowed us to examine the effect of ORF35 deletion on AngHV-1 infection of the host via a route mimicking natural infection. Eels were inoculated by immersion in water containing 4000 pfu/mL of the UK Luc or UK Luc ORF35 Del strain (Figure 3, middle column). Eels were analyzed by IVIS at 2, 4, 6, 8, and 10 dpi. As with the IP inoculation, a statistical analysis (taking each organ separately) revealed that in all organs, there were significant differences between strains both in terms of signal intensity and the number of positive subjects. As observed with IP inoculation, these significant differences between strains were the result of the lower or no signal expressed by the UK Luc Del ORF35 strain. Strikingly, except for a single fish that tested positive on 2 dpi (low signal and observed only in the gills), no other positive signals were observed among the UK Luc ORF35 Del group when inoculated by immersion. By contrast, for the UK Luc group, at least half of the subjects tested positive in all organs at 6 dpi, and 100% tested positive in all organs at 10 dpi.

The social interactions between eels represent a major transmission route for AngHV-1 between hosts, specifically through biting (Delrez et al., manuscript submitted). Consequently, we aimed to mimic this mode of transmission through intradermal inoculation using an electronic tattooing device. Eels were then analyzed by IVIS at 1, 2, 4, and 6 dpi. Taking each organ separately, the statistical analyses revealed that in all organs, the time post-infection and the strain had no significant impact on the signals and number of positive subjects, although the signals expressed by the UK Luc strain remain higher than UK Luc ORF35 Del strain in the skin at 4 and 6 dpi. Fish infected with either the UK Luc or UK Luc ORF35 Del strains showed positive signals at 2 dpi but only in the skin. Very low positive signals, close to the cut-off, were also detected in the gut–liver at 2 dpi, but there were confined to UK Luc ORF35 Del, but notably, no UK Luc ORF35 Del positive signals were detected at later timepoints in this organ. While the results provide some evidence that eels intradermally inoculated with either UK Luc or UK Luc ORF35 Del gradually develop a systemic infection, no positive signals were observed in the brain at any timepoint for UK Luc ORF35 Del. As expected, the skin showed the strongest signals, and for both strains, the signal was mainly co-localized at the intradermal inoculation sites. However, these signals reduced after 2 dpi in eels infected with UK Luc ORF35 Del (Figure 3B, right column), which corresponded to the healing and eventual disappearance of lesions at the site of inoculation.

Representative images of IVIS data (skin, gills, and brain) are presented in Figure 3C. Eels with the closest scores to the mean of each infection route were selected for image illustration.

Together, the results of these in vivo experiments demonstrate that AngHV-1 ORF35 is an essential virulent factor. Deletion of this gene led to an abortive replication cycle even after artificial inoculation by IP or intradermal injections. Of note, the inoculation of eels by immersion in water containing the virus led to no detectable infection by IVIS in all but one subject.

### 3.4. Testing of the Potential of the UK ORF35 Del Strain as a Vaccine Candidate for the Mass Vaccination of eels by Immersion in Water Containing the Virus

In the last section of this study, we tested the potential of the UK ORF35 Del strain as a vaccine candidate for the mass vaccination of eels by immersion in water containing the virus. The ability of this strain to induce a protective immune response against a wild-type challenge was tested using IVIS as follows (Figure 4). Naïve eels were first vaccinated by immersion in water containing different concentrations of UK ORF35 Del (100,000, 75,000, 50,000, 25,000, or 5000 pfu/mL of water), along with a group of eels exposed to psoralen/UV inactivated of the highest dose tested (corresponding to 100,000 pfu/mL of virions prior to inactivation). Psoralen/UV inactivation relies on a DNA crosslink affecting DNA replication and transcription but preserving the structure and the proteins of virus particles and so the ability of the virus to bind and enter host cells [29]. A group of eels were also mock-infected (Figure 4, Mock) at the time of primary infection. At 36 days post-exposure to the primary inoculation by immersion, the eels were challenged with the AngHV-1 UK Luc strain (4000 pfu/mL) and analyzed by IVIS at 3, 6, 9, 12, and 15 dpi (Figure 4).

Eels that were mock-infected or infected with Psoralen/UV-inactivated UK ORF35 Del at the time of primary inoculation and challenged expressed a normal kinetic of infection (Figure 4B, two right-end columns of each organ section). On day 3 post-challenge, approximately half of the eels expressed a bioluminescent signal in the skin and/or the gills, with only one positive eel in the internal organs (brain and heart). At 6 dpi and at later timepoints, all the eels from both groups (mock and Psoralen/UV-inactivated groups) expressed infection in the skin and the gills but also in the other organs tested (brain, heart, and gut–liver). These data demonstrate that the challenge used in this study led to the systemic and comparable infection of all non-vaccinated subjects and subjects vaccinated with the Psoralen/UV-inactivated virus.

Primary exposure to infectious particles of the UK ORF35 Del strain resulted in a reduced replication of the UK Luc challenge strain in a dose–effect manner (Figure 4B). Statistical analyses of each organ across all the timepoints between groups were performed to compare the radiance signal (*p*(*rad*)) and number of positive fish (*p*(*no*)) (Figure 4B). These analyses demonstrated that all tested doses of infectious particles of the UK ORF35 Del strain led to a significant reduction in the number of positive fish. This conclusion was reached after considering all the organs tested. A statistical analysis of signal radiance also supported the ability of the UK ORF35 Del strain to induce a protective immune response against a wild-type challenge. A significant effect was observed for all doses tested for the skin and the gills, while a significant effect was observed for the brain, the heart, and the gut–liver at doses of 50,000 pfu/mL or higher. When considering isolated timepoints and specific organs, it appeared that the lower doses of UK ORF35 Del tested were associated with few fish expressing infection by the challenging virus (see, for example, doses of 5000 and 25,000 pfu/mL for the brain at day 15). These results suggest that lower doses could reveal a more pronounced dose–effect correlation.

Together, the results of these experiments support the potential of the UK ORF35 Del strain as a candidate vaccine against AngHV-1 for the mass vaccination of eels by immersion in water containing the infectious virus. Of note, vaccination of the fish at the doses of 100,000 and 50,000 pfu/mL led to no detection of positive fish (independently of the time post-challenge or the organ tested). At the dose of 75,000 pfu/mL, only three fish were detected as slightly positive (day 9, heart; day 12, gills and brain).

The results presented in Figure 3 (abortive infection after inoculation by immersion in infectious water) and in Figure 4 (ability of the virus to confer a protective immunity against a wild-type challenge) suggest that the vaccination effect of the UK ORF35 Del strain could be independent of the infectivity of the virus, acting like an inactivated vaccine. The results obtained with the fish vaccinated with psoralen-UV-inactivated particles (Figure 4B, Psoralen/UV, 100,000 pfu/mL) of UK ORF35 Del excluded this hypothesis. The fish of this group expressed the infection by the wild-type UK Luc strain comparably to the mock-vaccinated group. No statistical differences in terms of the radiance signal or number of positive fish were observed between these two groups in any of the organs tested. Representative images of IVIS data are presented in Figure 4C. Eels with the closest scores to the mean of the selected groups were selected for image illustration.

Taken together, the results of this study demonstrate that the deletion of the ORF35 of AngHV-1 does not affect the production of virions in a cell culture but induces an abortive infection in vivo after inoculation by immersion in water containing the virus. Surprisingly, we observed that this abortive infection was essential and sufficient to induce a protective immune response against a wild-type challenge.

## 4. Discussion

To date, despite its negative impact on eel aquaculture and wild eel populations, there is no effective vaccine against AngHV-1 available on the market. Control of AngHV-1 disease by shifting water temperatures below 22 °C to reduce lytic viral replication and associated clinical symptoms, including mortalities, can reduce the impact of the disease. However, it has the disadvantage to generate latently infected carriers that would inevitably reactivate the infection later in life, causing the re-emergence of disease symptoms and a transmission of the virus to naïve subjects [30]. This may be especially detrimental when farmed eels are used in restocking programs of wild habitats. Consequently, there is a need for a safe and efficacious vaccine against AngHV-1 that is compatible with the mass vaccination of eels. Live attenuated vaccines represent the favored option for the development of a vaccine compatible with mass vaccination. The rational design of the development of an attenuated virus vaccine relies on the identification of non-essential viral genes playing a key role in virulence. A major barrier towards this process with AngHV-1 is the fact that there have been, to date, no studies conducted on the function of any of its genes.

Here, based on the results published on its orthologue encoded by CyHV-3, we identified and tested AngHV-1 ORF35 as a potential gene candidate for the production of a live attenuated recombinant vaccine [18,19,20]. Analyses of ORF35-deleted recombinants led to the following observations: (i) AngHV-1 ORF35 is not essential for viral growth in cell culture, and its deletion does not affect the production of extracellular virions despite reducing the size of the viral plaque. (ii) AngHV-1 ORF35 is an essential virulence factor, and its deletion led to abortive infection in vivo. (iii) Inoculation of an infectious AngHV-1 ORF35-deleted recombinant by immersion induced a protective immune response against a wild-type challenge in a dose-dependent manner. This study suggests that AngHV-1 ORF35 has singular functions compared to its orthologues encoded by CyHV-2 and CyHV-3. It also supports the potential of AngHV-1 ORF35-deleted recombinants for the mass vaccination of eels by immersion.

Orthologues of AngHV-1 ORF35 are encoded by other Cypriniviruses (ORF57 of CyHV-1, -2, and -3). Including the results of the present study, the impact of the deletion of these orthologues on virus replication has been tested in cell culture and in vivo for three cypriniviruses: CyHV-3 [18,19], CyHV-2 [21], and AngHV-1 (present study). These studies identified consistently these orthologues as non-essential genes for replication in cell culture. However, they also suggested that these orthologues encode different functions or at least functions of different importance in the biological cycle of these viruses. This conclusion is supported by observations performed both in vitro and in vivo. In vitro deletion of CyHV-3 ORF57 induced a drastic reduction in virion production in cell culture, while the deletion of the orthologue had no impact on CyHV-2 [21] or AngHV-1 (Figure 2C,E). Similarly, in vivo experiments revealed different phenotypes, with deletion of the orthologue in CyHV-3, CyHV-2, and AngHV-1 inducing strong attenuation, marginal attenuation, and abortive infection, respectively. Different non-exclusive hypotheses could explain the different phenotypes observed among cypriniviruses. The first obvious hypothesis is that the different orthologues acquired different functions since divergence from their common ancestor. A second hypothesis could be that the functions mediated by the orthologues could be redundant in some viruses (with deletions compensatedby other viral proteins) while being non-redundant and, therefore, essential in other viruses. According to this hypothesis, the deletion of the orthologue could mediate either no/minor phenotypes or a strong phenotype. Other hypotheses could rely on host-cell factors rather than on functional redundancy or functional diversity among viral genes. For example, it could be possible that in certain hosts, some cellular protein(s) can trans-complement the functions of deleted viral orthologues. Finally, assuming that the AngHV-1 ORF35 orthologues are proteins involved in the immune evasion of host innate immunity, the different phenotypes observed in vitro and in vivo could reveal the absence or variable importance of the innate immune component targeted by the viral orthologues in the different host models. Further studies are required to unravel the functions of the orthologues of AngHV-1 ORF35. These projects of comparative virology are likely to unravel fascinating aspects of host-virus evolution.

The present study revealed the potential of AngHV-1 ORF35-deleted recombinants as a vaccine candidate for the mass vaccination of eels by immersion. Surprisingly, despite the abortive infection observed after inoculation by immersion in infectious water, the protective immune response induced by the vaccine was shown to be dependent on virus infectivity and subject to a dose–effect relationship. These results suggest that the abortive infection is essential for antigen presentation and/or stimulation of the innate immune response to induce the protection observed. The abortive infection observed in vivo for the AngHV-1 ORF35-deleted recombinant is surprising for two reasons. First, because deletion of the orthologue of AngHV-1 ORF35 in CyHV-2 and CyHV-3 led, respectively, to mild attenuation and strong attenuation but not to abortive infection [18,21]. Second, because deletion of the ORF35 gene had no negative impact on AngHV-1 virion production in a cell culture (Figure 2C,E). Some of the hypotheses discussed above may consolidate the results observed in vitro and in vivo for the AngHV-1 ORF35-deleted recombinant. The dispensable nature of ORF35 for efficient replication in EK-1 cells, while it is essential for infection in vivo, could manifest the expression in EK-1 cells but not in host cells in vivo of a cellular protein trans-complementing the deletion of ORF35. A second and preferred hypothesis could be that ORF35 is essential in vivo to block an innate antiviral immune mechanism that is not functional in EK-1 cells. Experiments are currently ongoing to address these hypotheses.

The candidate vaccine developed in this study is compatible with the mass vaccination of eels by immersion. It could be used to control AngHV-1 infection both in the context of eel aquaculture and eel conservation programs. Due to their complex lifecycle, anguillid eels cannot be bred in captivity. Consequently, all anguillid eels produced by the aquaculture sector or used in restocking programs of wild habitats rely on wild-caught stock of glass eels. Recent studies performed on European eels suggested that glass eels enter estuaries free of AngHV-1 and then become infected in fresh water when they mature to the yellow and silver stages [31]. The present study was performed on yellow eels. It will be interesting to determine, in future experiments, whether the candidate vaccine developed in this study can be used to vaccine glass eels successfully by immersion. Of note, glass eels have been shown to be immune-competent and able to mount an adaptive immune response after vaccination [32]. If successful, vaccination could be applied to glass eels maintained in facilities free of AngHV-1, before their transfer after the onset of immunity to farms and to a wild habitat. In the latter case, we hope that the vaccination could contribute to an increase in the number of eels that successfully reach a spawning area and reproduce by reducing the negative impact of AngHV-1 on endangered eel species.

## 5. Conclusions

Here, we investigated using recombinant strains and bioluminescent imaging the effect of AngHV-1 ORF35 deletion on virus replication in vitro, virulence in vivo, and the potential of AngHV-1 ORF35-deleted recombinants as vaccine candidates for the mass vaccination of eels by immersion. The results suggest that the AngHV-1 ORF35 expresses singular and essential properties in vivo compared to its orthologues encoded by CyHV-2 and CyHV-3. They also support the potential of AngHV-1 ORF35-deleted recombinants as vaccine candidates for the mass vaccination of eels by immersion.

## Data Availability

All the data are included in this article. The uncropped images used to build the figures in the paper are provided as Supplementary Materials. Further inquiries can be directed at the corresponding author.

## Supplementary Materials

The following supporting information can be downloaded at: https://www.mdpi.com/article/10.3390/vaccines12121423/s1, Figure S1: Uncropped original images used to build the figures of this paper; Table S1: Oligonucleotide primers used this study.
