# Supplementary material for: Development Using Bioluminescence Imaging of a Recombinant Anguillid Herpesvirus 1 Vaccine Candidate Associated with Normal Replication In Vitro but Abortive Infection In Vivo
Source: Vaccines (Basel). 2024 Dec 17;12(12):1423. doi: 10.3390/vaccines12121423 (PMC11728778; doi:10.3390/vaccines12121423)

Figure S1. Uncropped original images used to build the figures of this paper.

Figure 2A images

cDNA

These gel images were cropped and used in the manuscript. The Red “X” indicates that these lanes were not used in the manuscript.

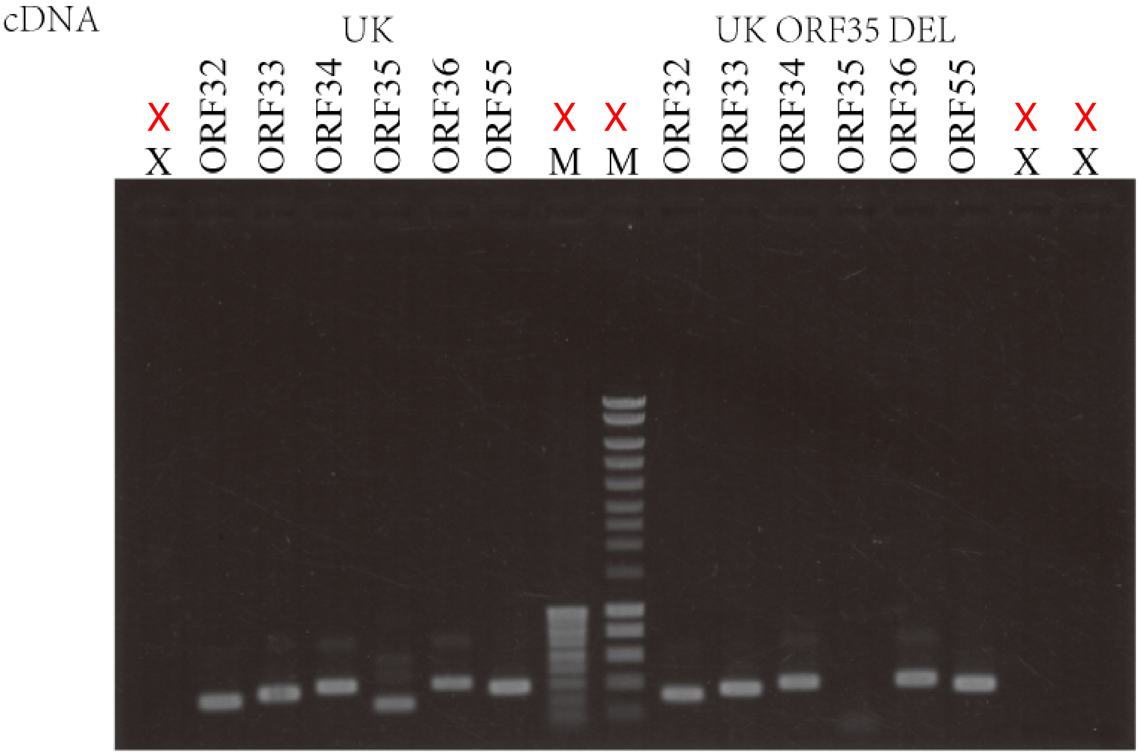

Figure 2A images

cDNA

These gel images were cropped and used in the manuscript. The Red “X” indicates that these lanes were not used in the manuscript.

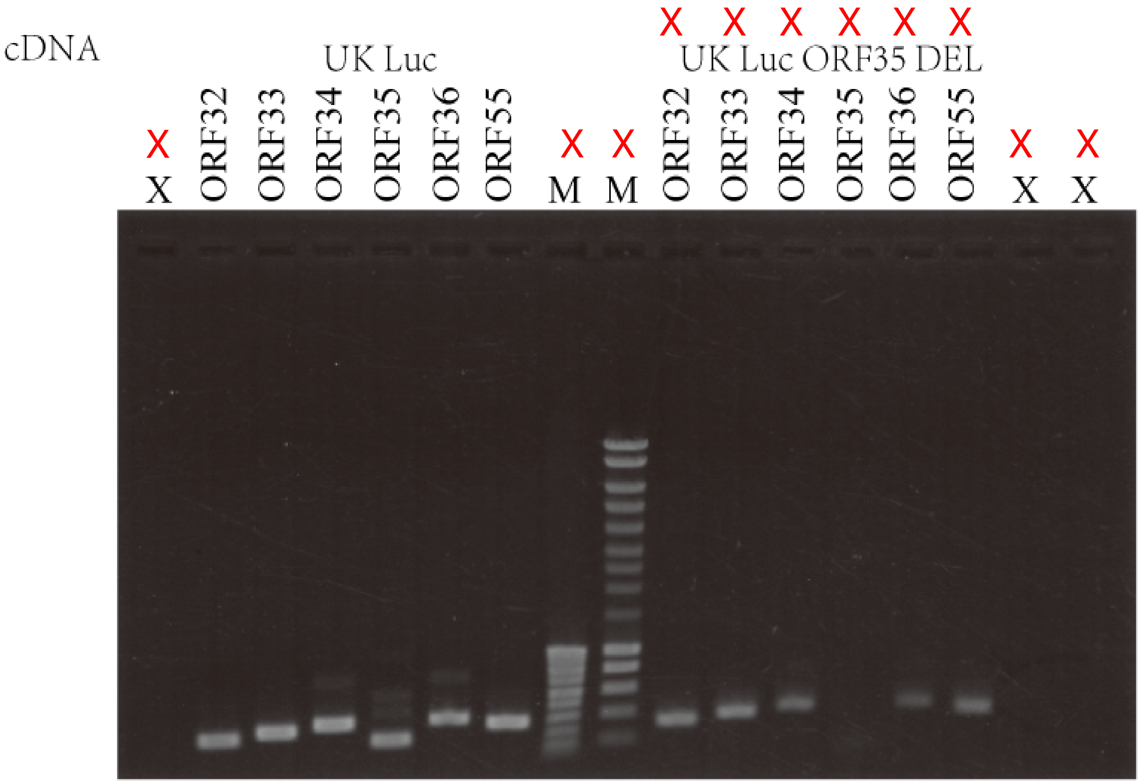

Figure 2A images

cDNA

These gel images were cropped and used in the manuscript. The Red "X" indicates that these lanes were not used in the manuscript.

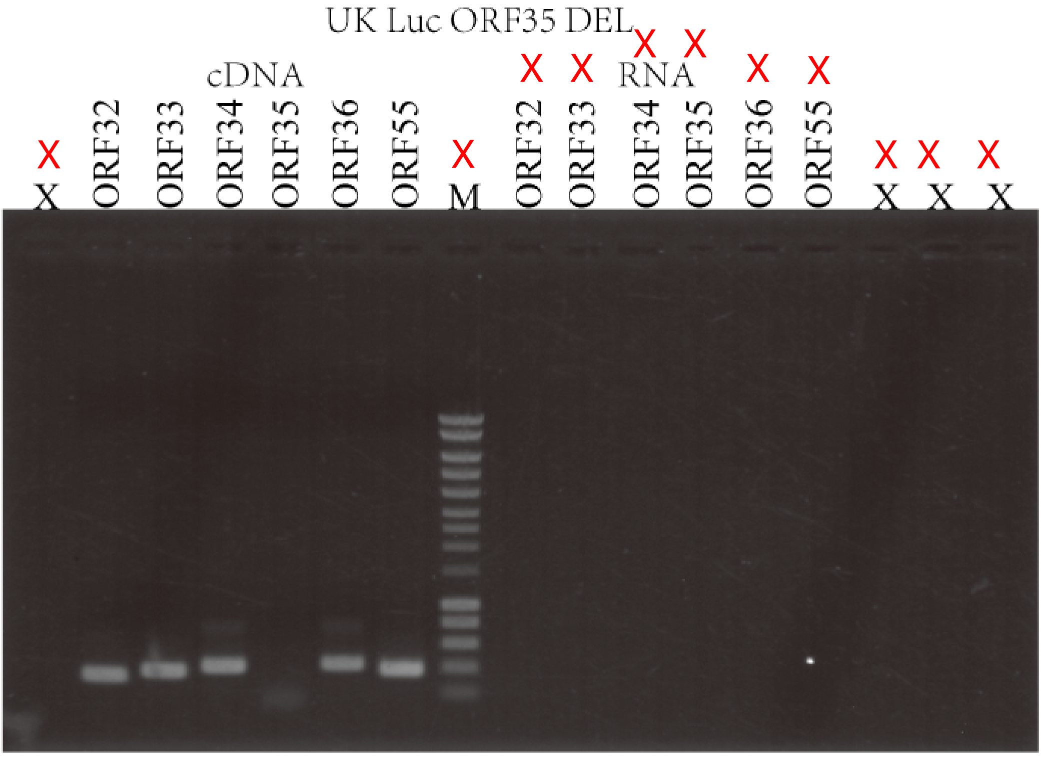

Figure 2B images

Bioluminescence

The yellow boxed well was selected to represent the selected group. The images were cropped in the manuscript.

UK

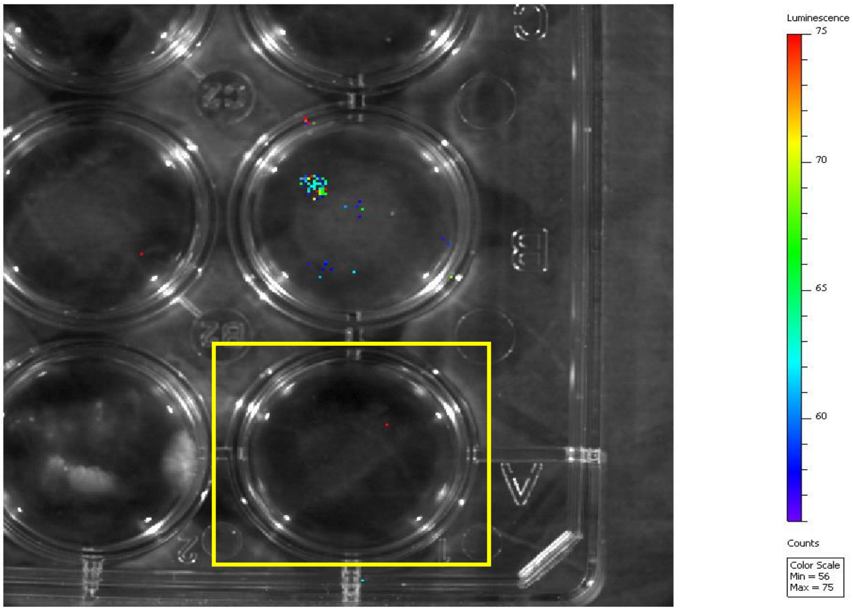

UK ORF35 Del

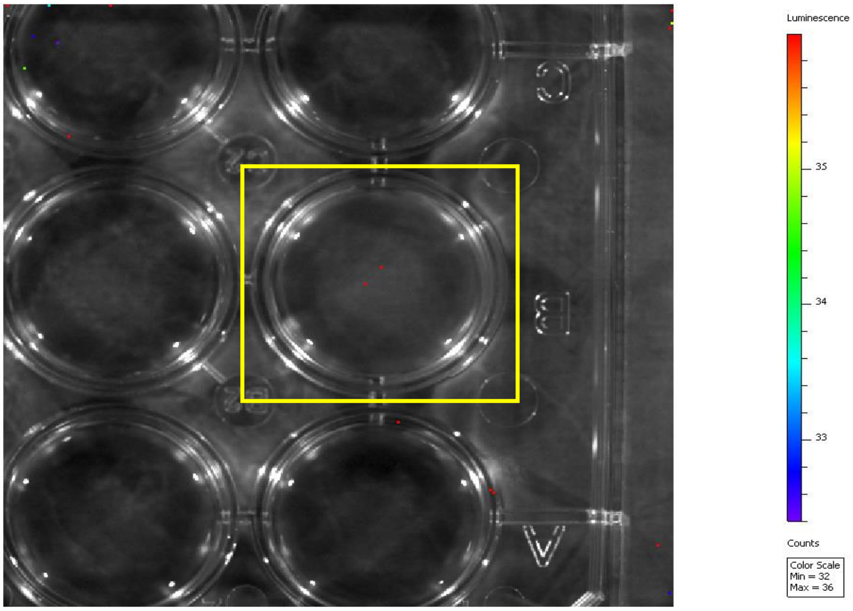

Figure 2B images

Bioluminescence

The yellow boxed well was selected to represent the selected group. The right end radiance was adjusted in the manuscript. The images were cropped in the manuscript.

UK Luc

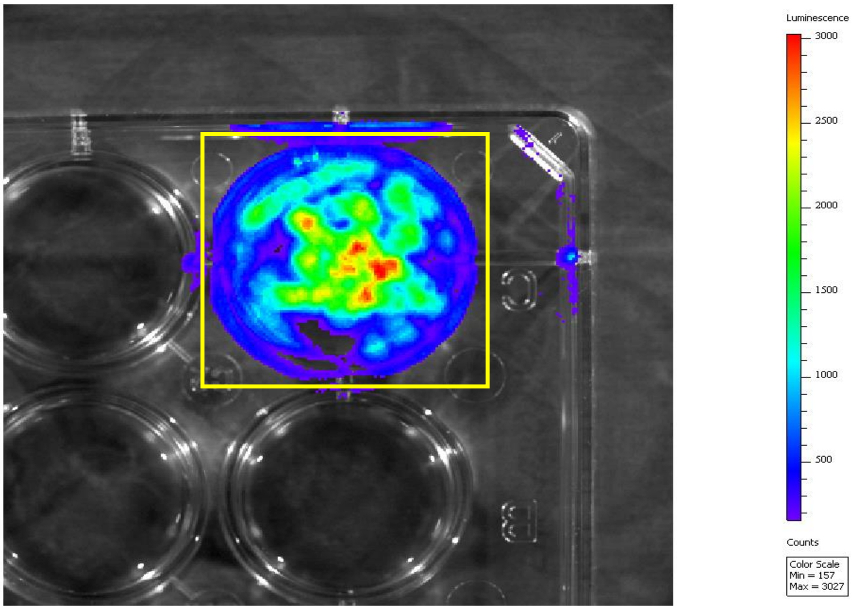

UK Luc ORF35 Del

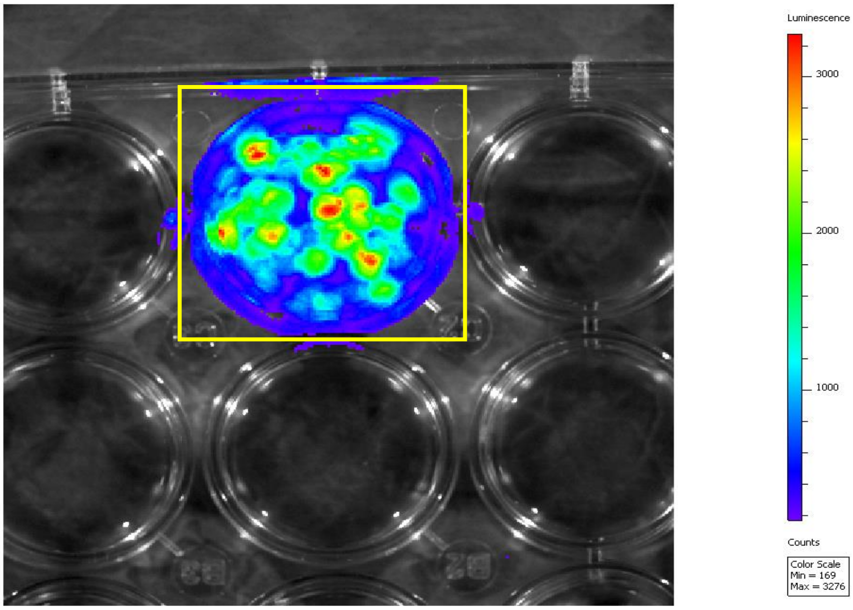

Figure 2B images

Epifluorescence

These images were used in the manuscript for the group UK.

DAPI

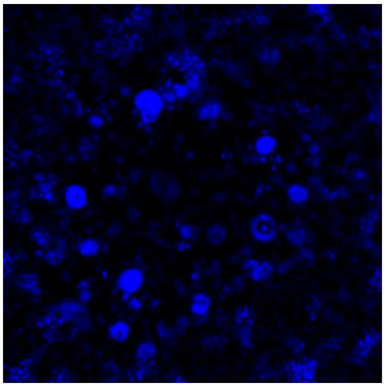

Anti-AngHV-1 or CopGFP

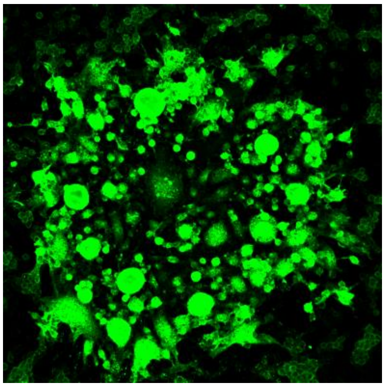

mCherry

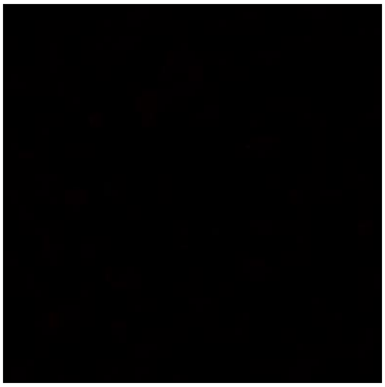

Overlay

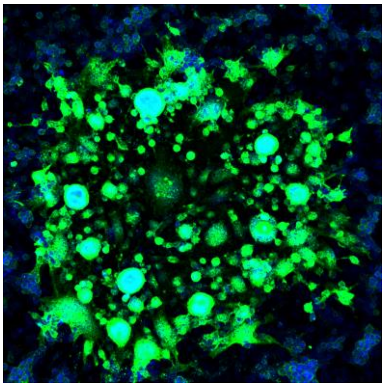

Figure 2B images

Epifluorescence

These images were used in the manuscript for the group UK ORF35 Del.

DAPI

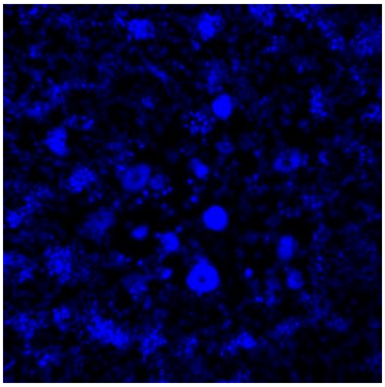

Anti-AngHV-1 or CopGFP

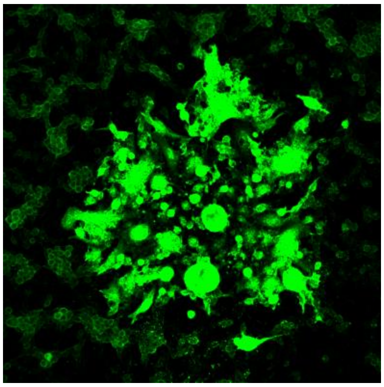

mCherry

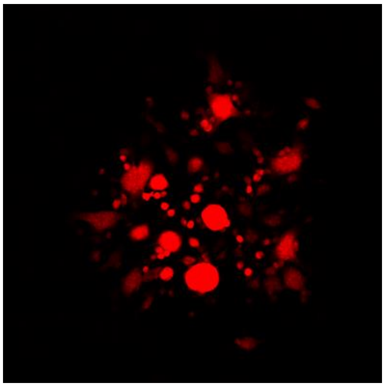

Overlay

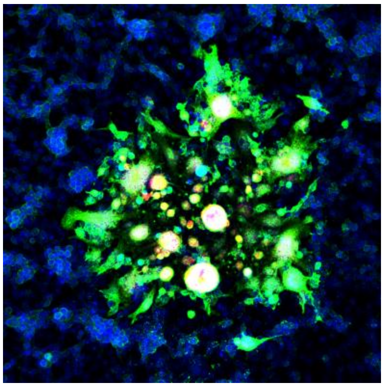

Figure 2B images

Epifluorescence

These images were used in the manuscript for the group UK Luc.

DAPI

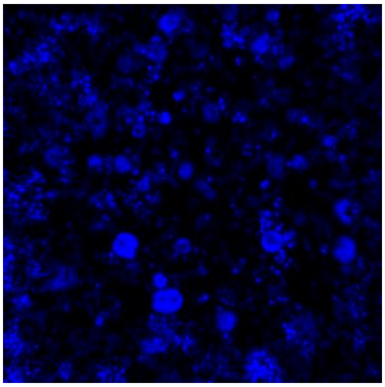

Anti-AngHV-1 or CopGFP

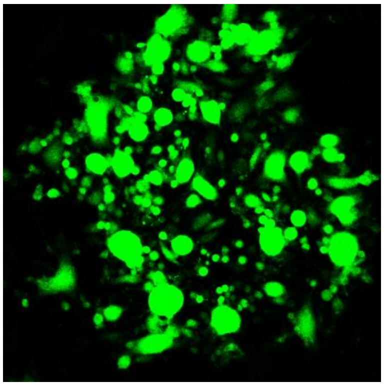

mCherry

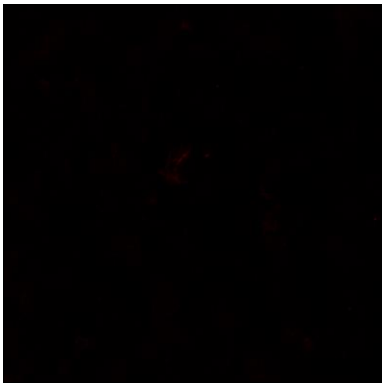

Overlay

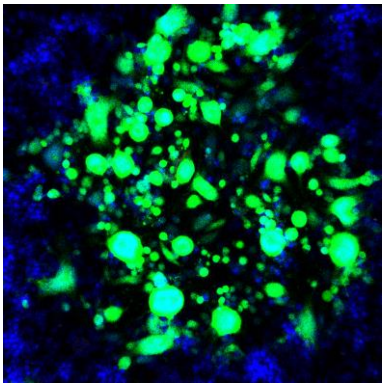

Figure 2B images

Epifluorescence

These images were used in the manuscript for the group UK Luc ORF35 Del

DAPI

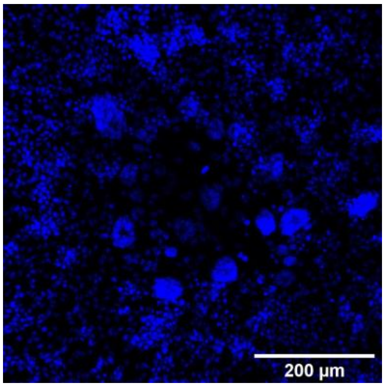

Anti-AngHV-1 or CopGFP

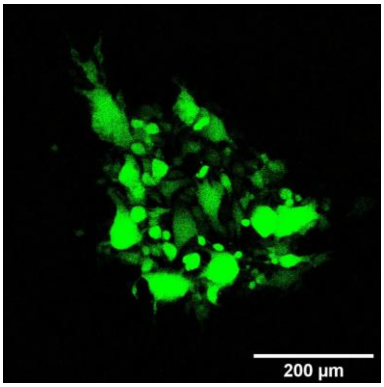

mCherry

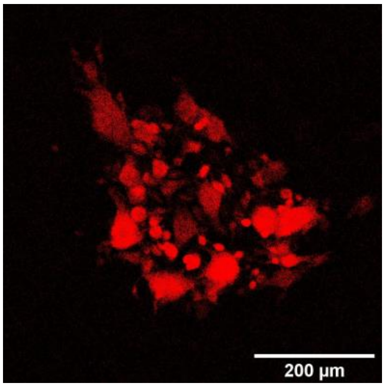

Overlay

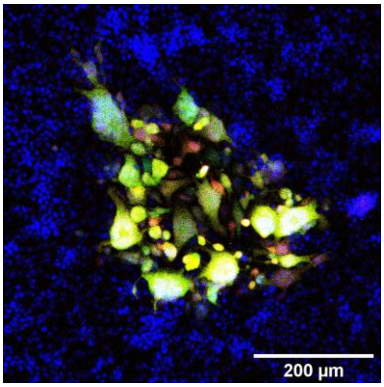

Figure 3C images

The yellow boxed eel skin, gills, brain were taken for representing the group. Eels with the closest scores to the mean of each infection route have been selected for illustration. The right end radiance scale was adjusted for each image The images were cropped in the manuscript.

Skin left image

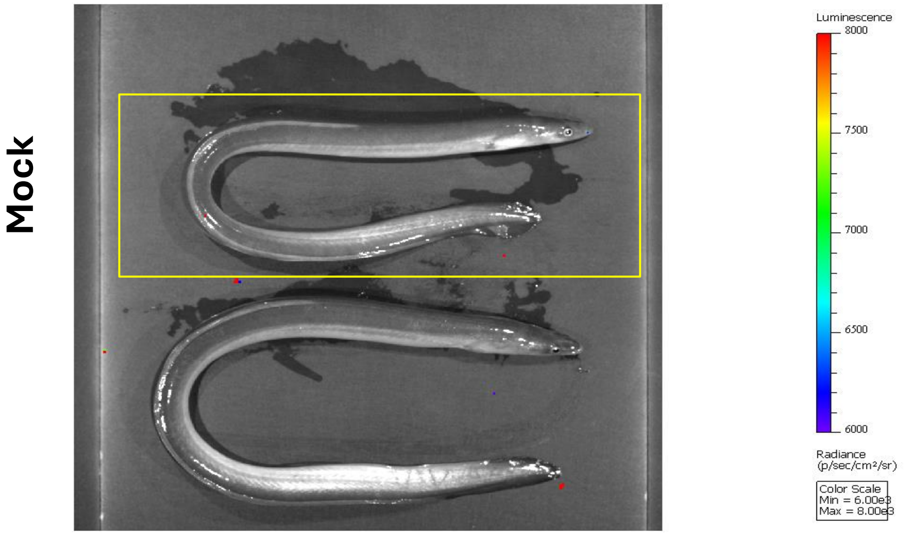

Skin right image

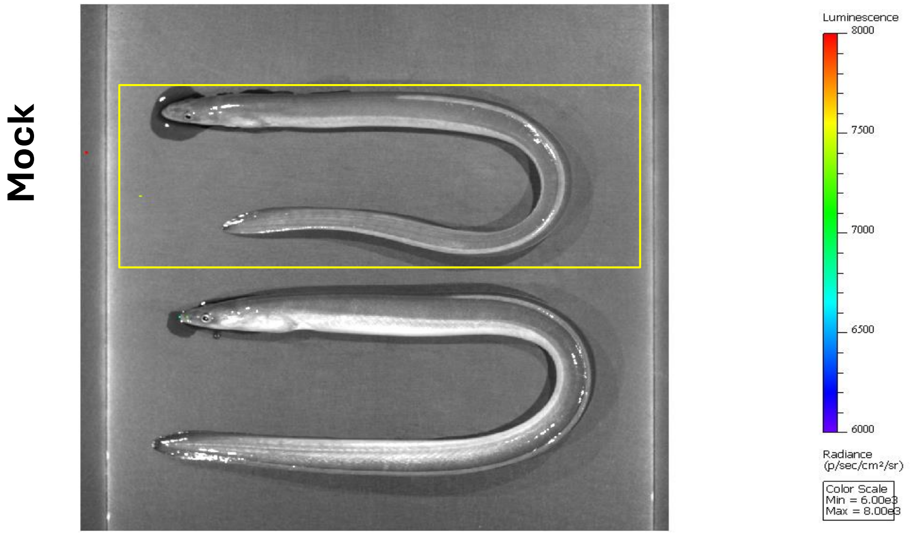

Figure 3C images

The gills were rotated clockwise or anti-clockwise depending on the gills position to maintain consistency with the other group gills. The gills and brain images were cropped in the manuscript.

Gills

Mock

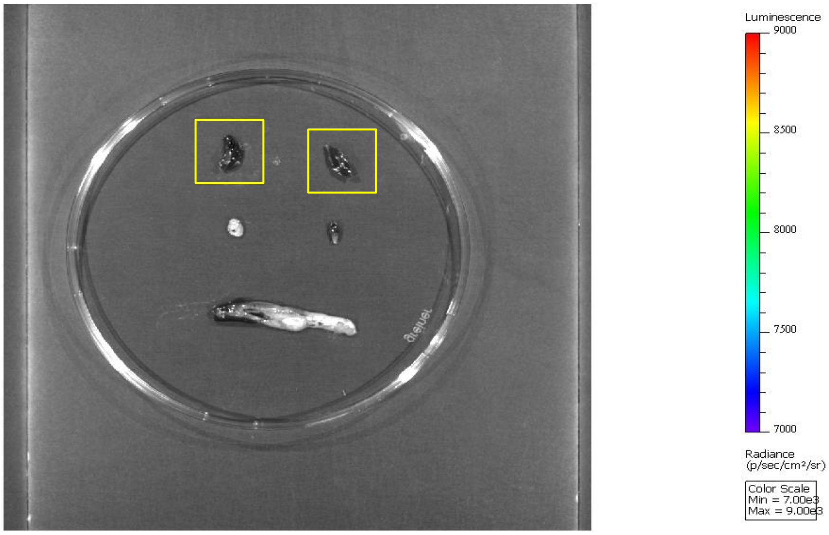

Brain

Mock

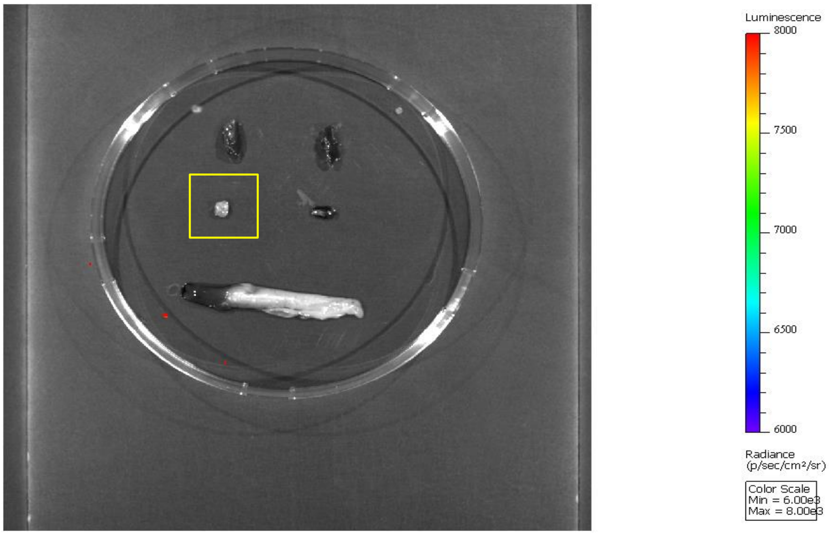

Figure 3C images

The images were cropped in the manuscript.

Skin left image

IP injection
UK Luc

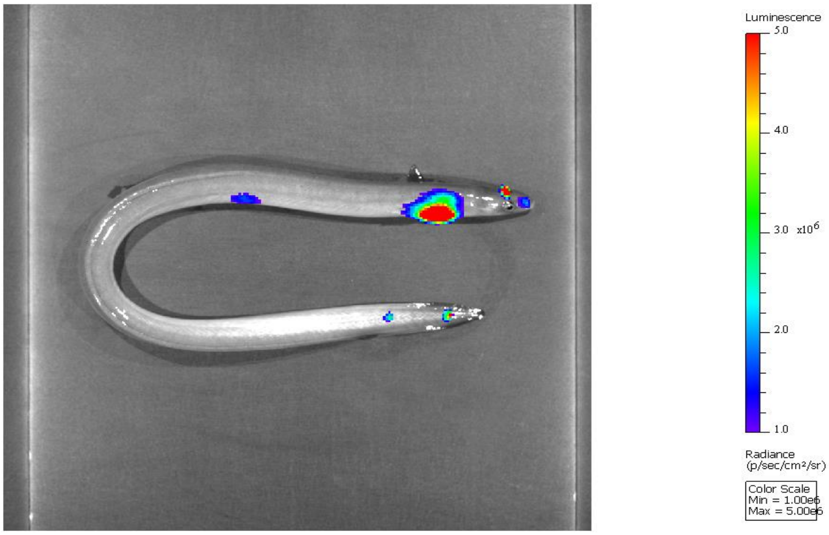

Skin right image

IP injection
UK Luc

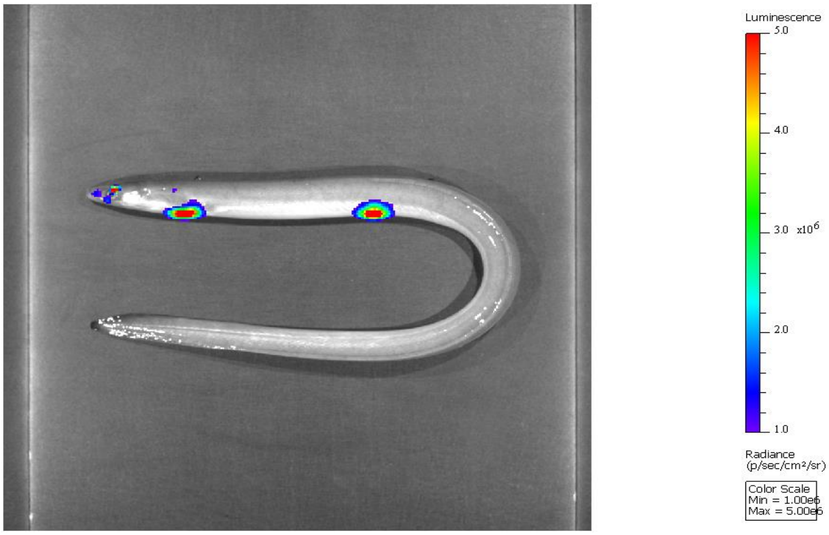

Figure 3C images

The yellow boxed gills, and brain were taken for representing the group. The right end radiance scale was adjusted. The images were cropped in the manuscript.

Gills

IP injection
UK Luc

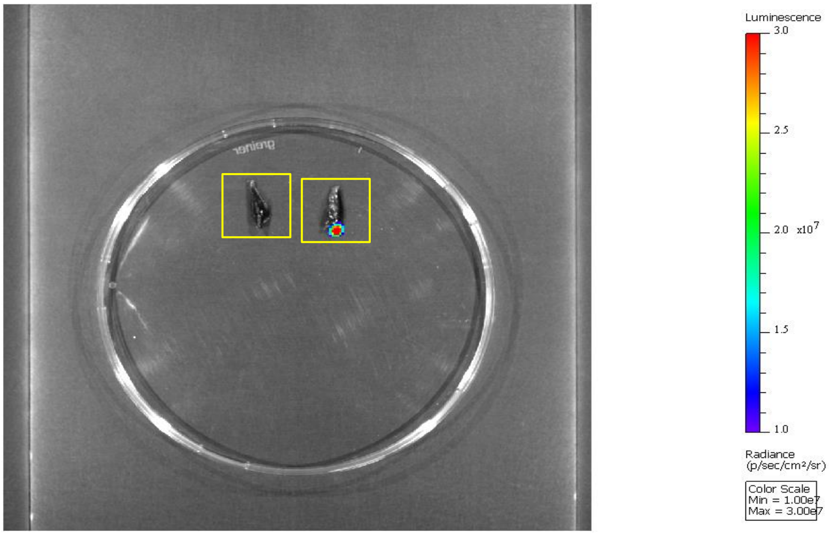

Brain

IP injection
UK Luc

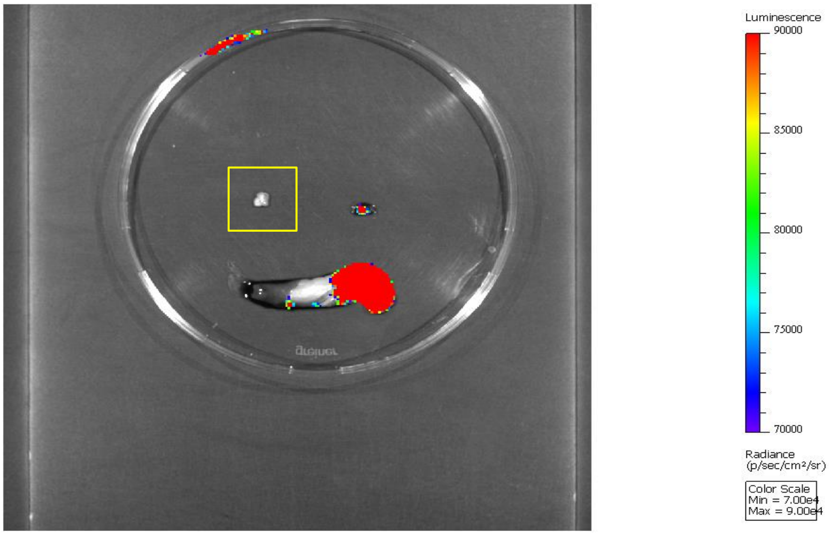

Figure 3C images

The images were cropped in the manuscript.

Skin left image

IP injection
UK Luc ORF35 DEL

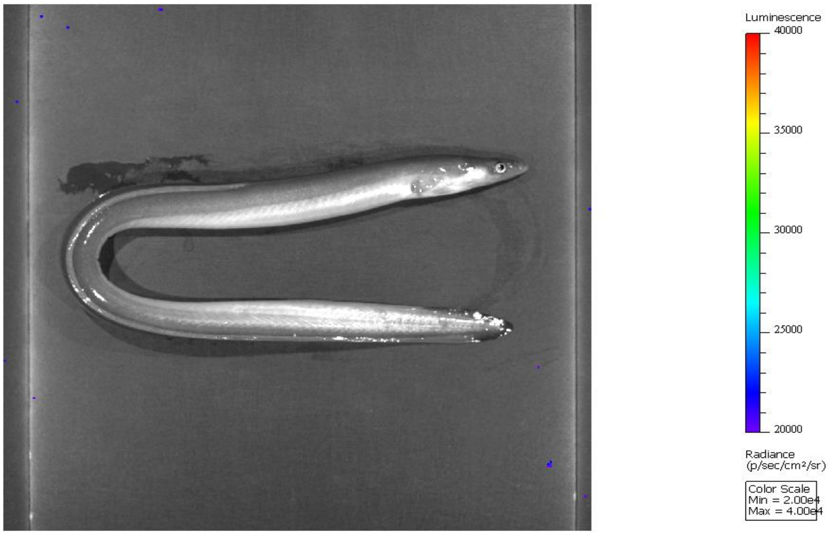

Skin right image

IP injection
UK Luc ORF35 DEL

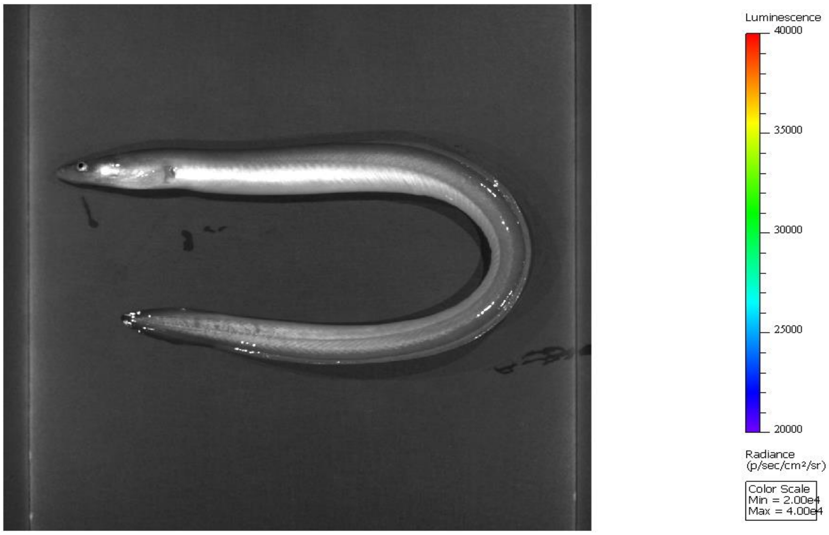

Figure 3C images

The yellow boxed gills, and brain were taken for representing the group. The right end radiance scale was adjusted. The images were cropped in the manuscript.

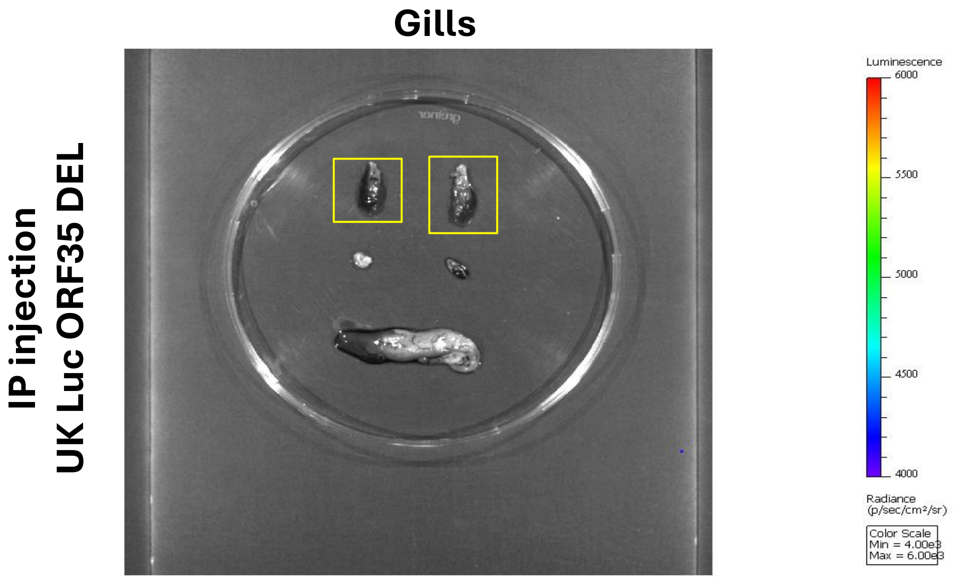

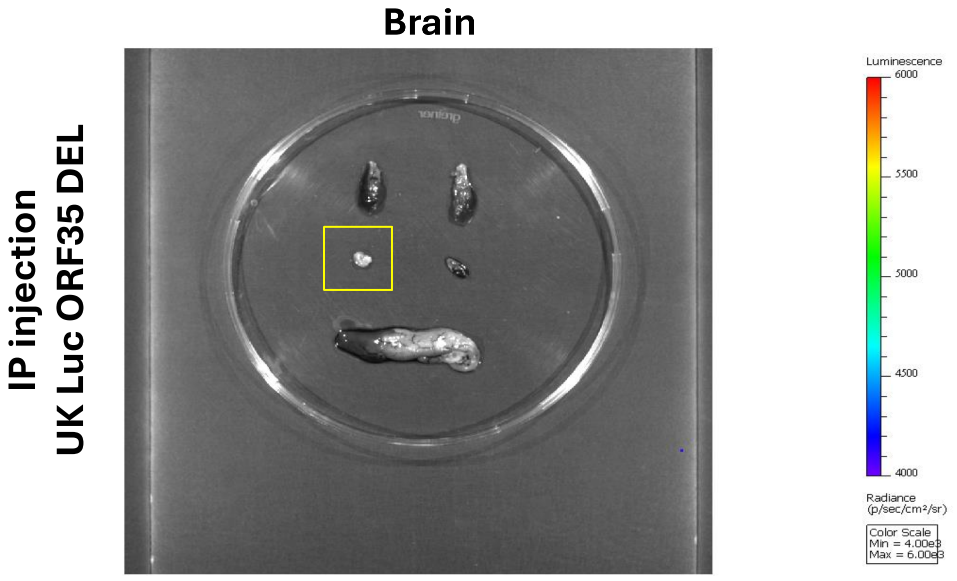

Figure 3C images

The images were cropped in the manuscript.

Skin left image

Immersion
UK Luc

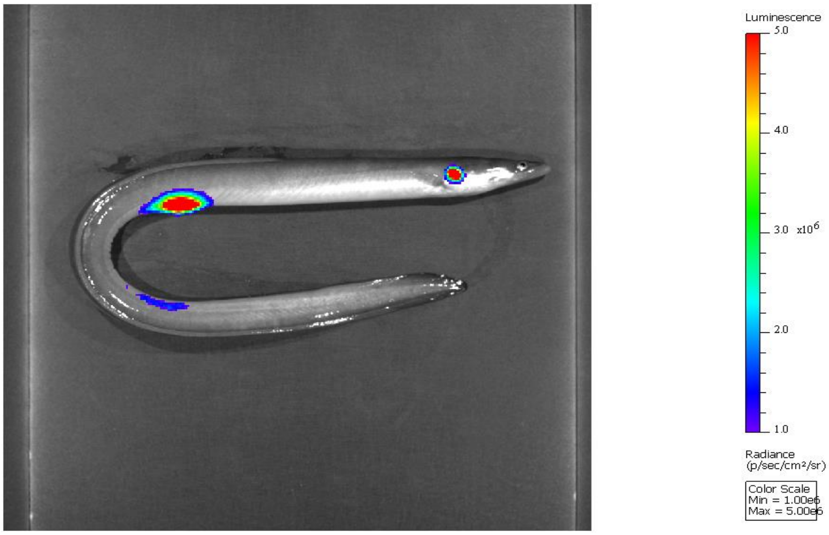

Skin right image

Immersion
UK Luc

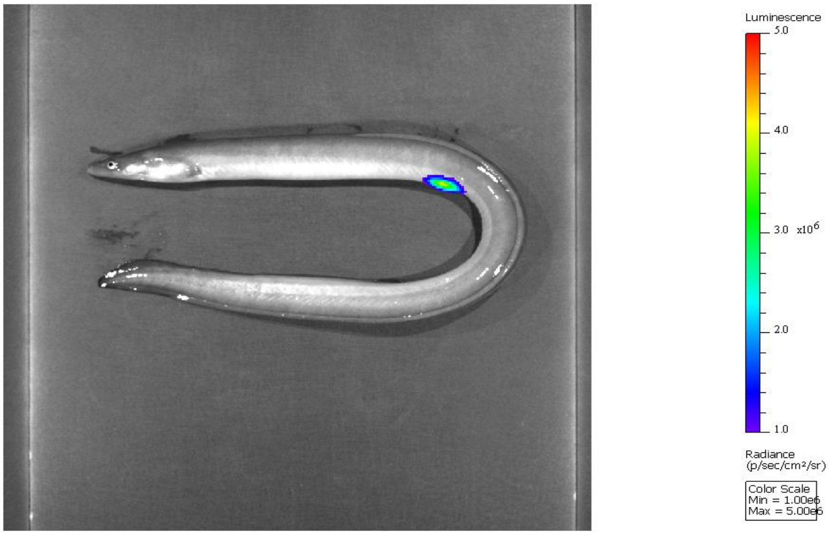

Figure 3C images

The yellow boxed gills, and brain were taken for representing the group. The right end radiance scale was adjusted. The images were cropped in the manuscript.

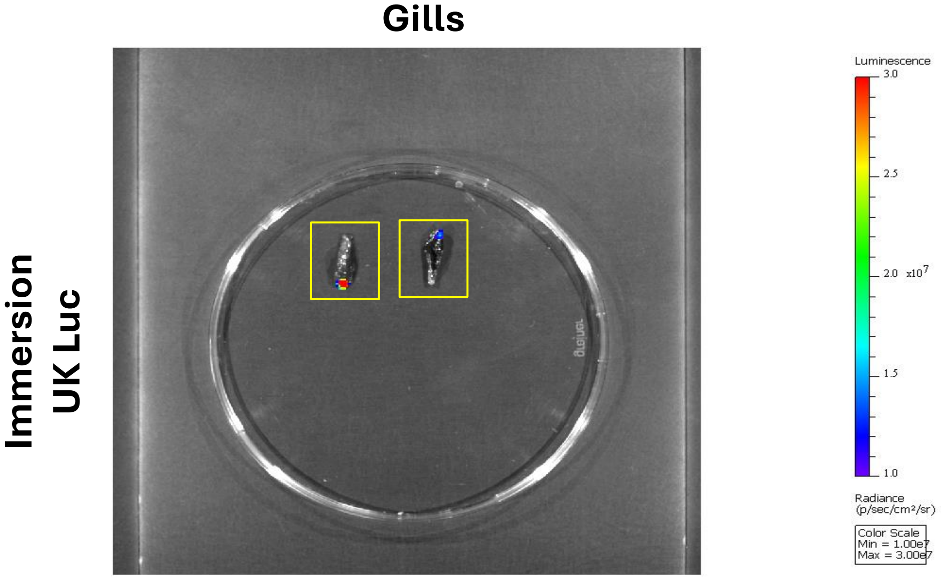

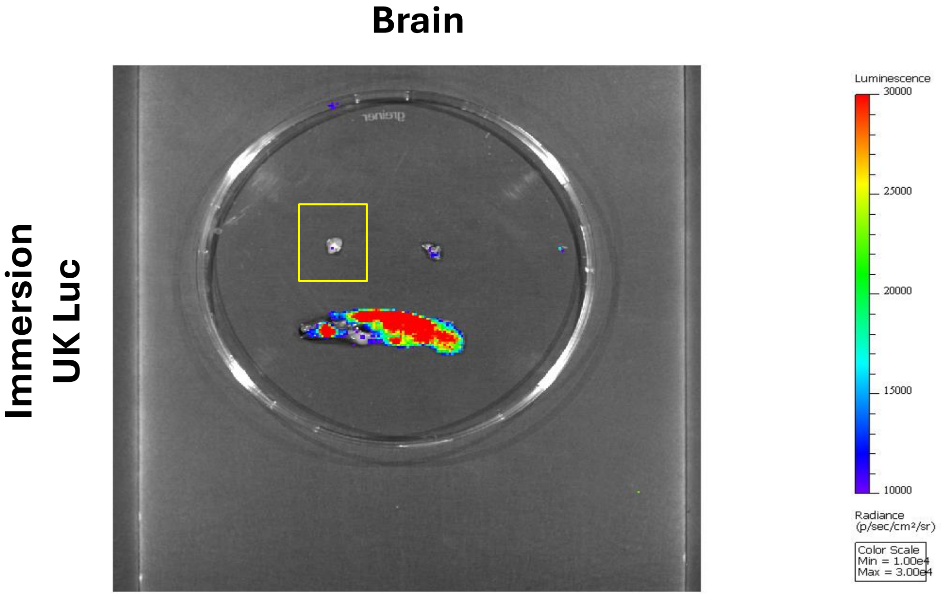

Figure 3C images

The images were cropped in the manuscript.

Skin left image

Immersion
UK Luc ORF35 Del

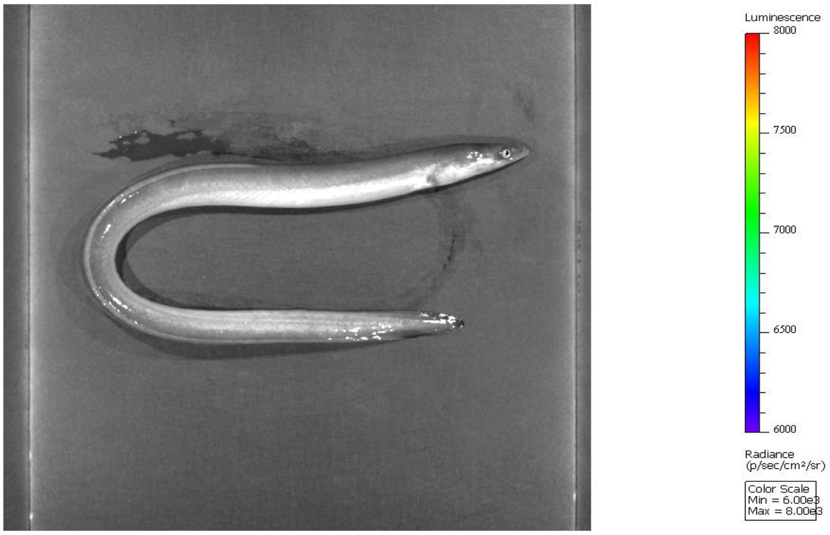

Skin right image

Immersion
UK Luc ORF35 Del

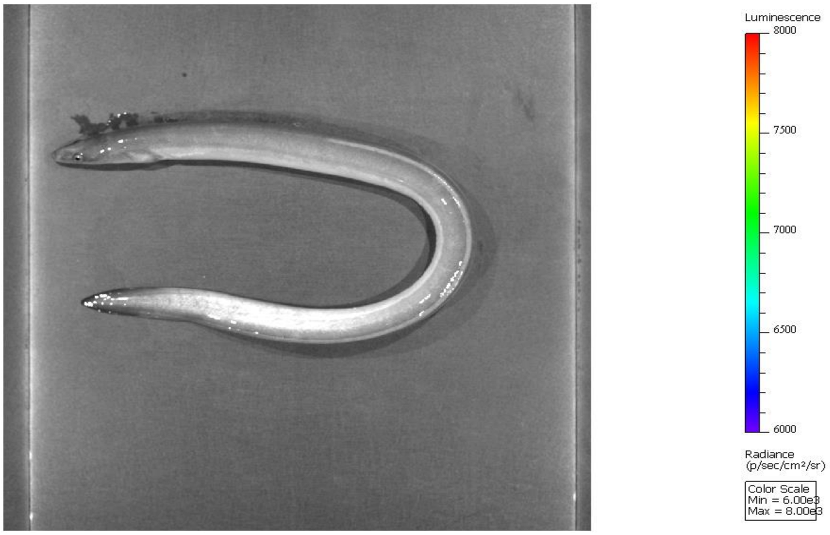

Figure 3c images

The yellow boxed gills, and brain were taken for representing the group. The right end radiance scale was adjusted. The images were cropped in the manuscript.

Gills

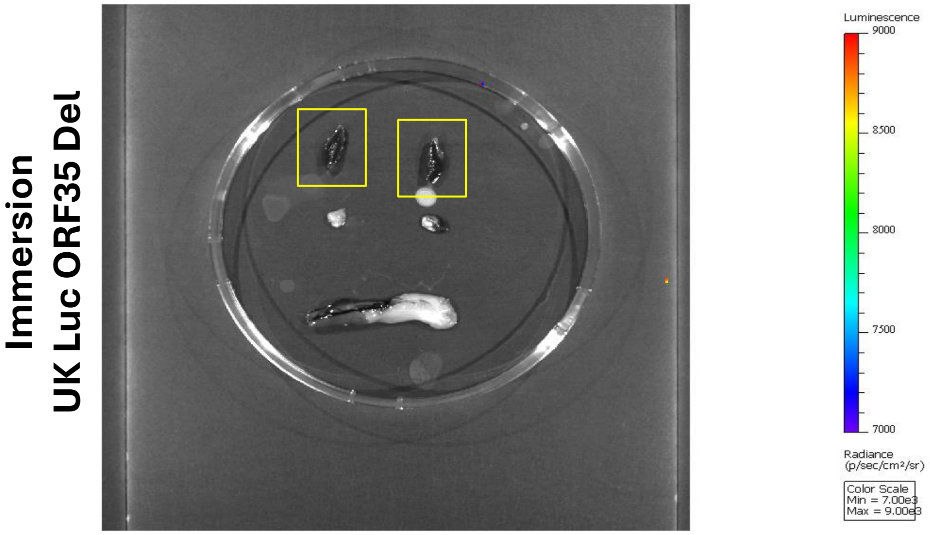

Brain

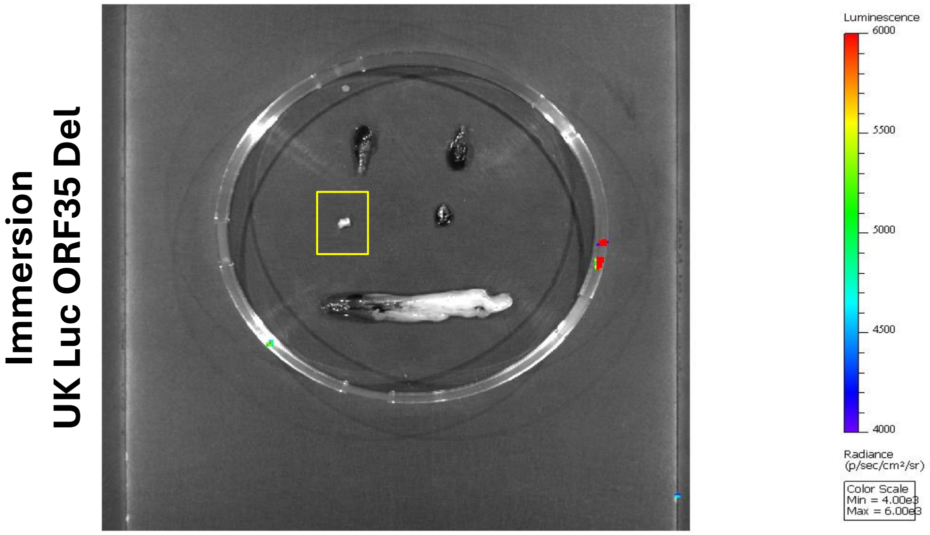

Figure 3C images

The images were cropped in the manuscript.

Skin left image

Intradermal
UK Luc

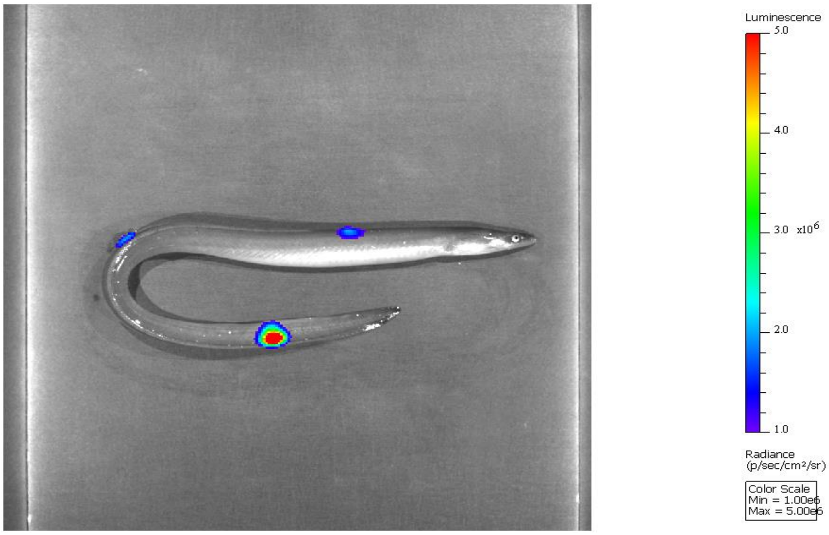

Skin right image

Intradermal
UK Luc

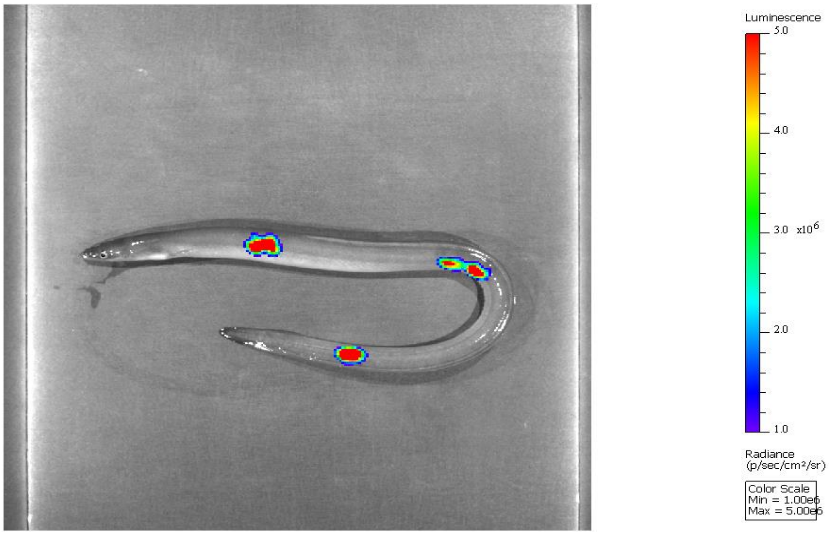

Figure 3C images

The yellow boxed gills, and brain were taken for representing the group. The right end radiance scale was adjusted. The images were cropped in the manuscript.

Gills

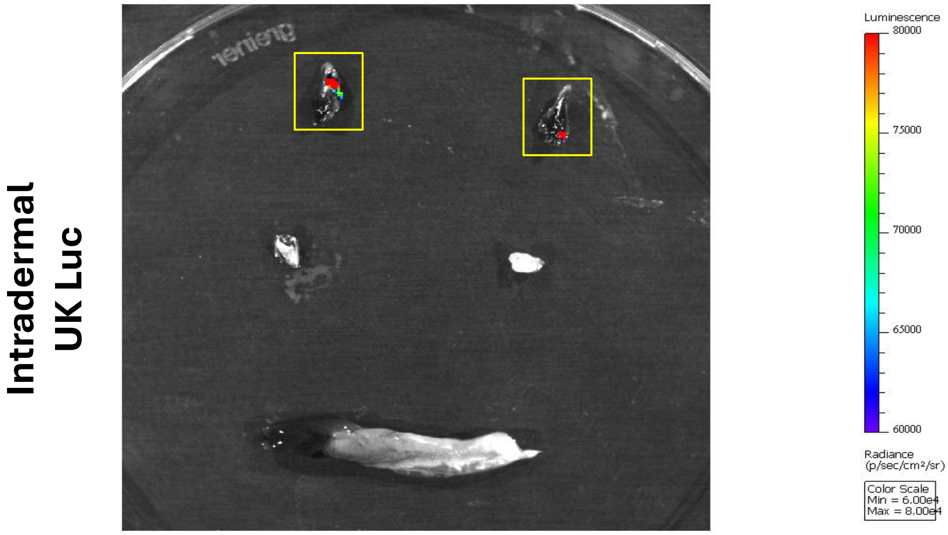

Brain

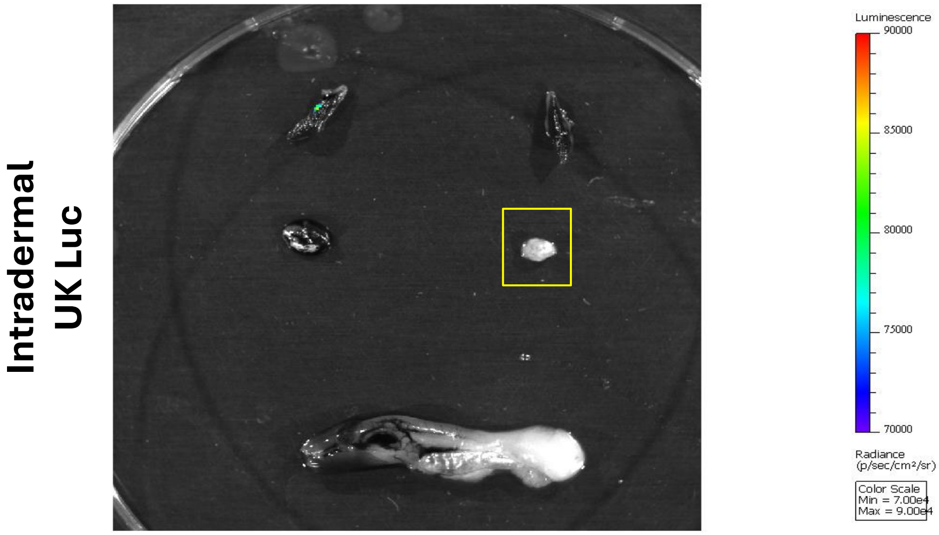

Figure 3C images

The yellow boxed eel skins were taken for representing the group. The images were cropped in the manuscript.

Skin left image

Intradermal
UK Luc ORF35 Del

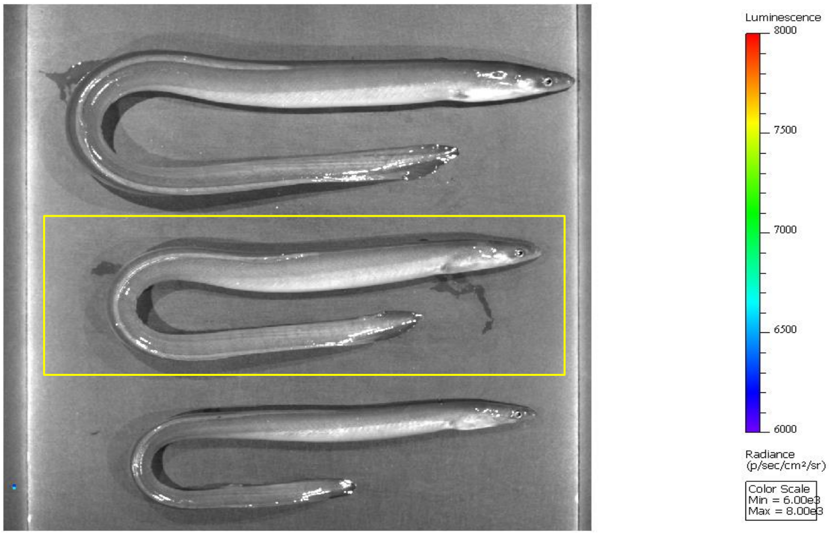

Skin right image

Intradermal
UK Luc ORF35 Del

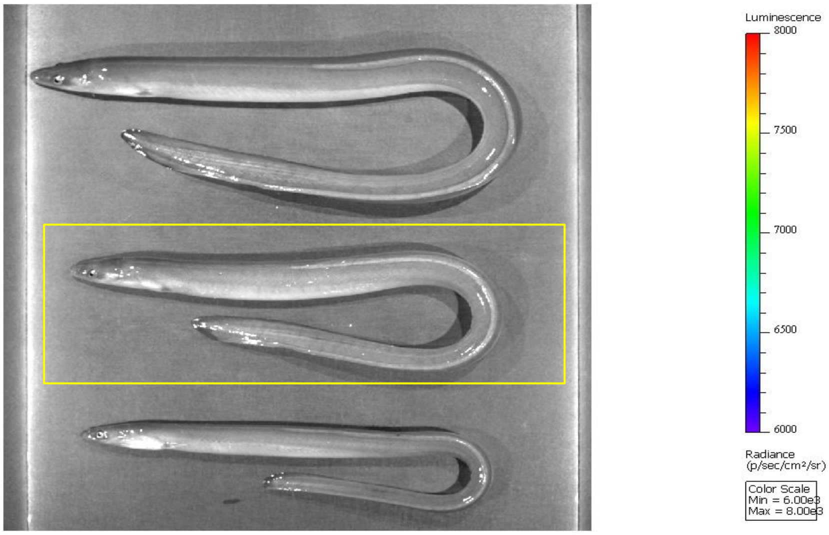

Figure 3C images

The yellow boxed gills, and brain were taken for representing the group. The right end radiance scale was adjusted. The images were cropped in the manuscript.

Gills

Intradermal
UK Luc ORF35 Del

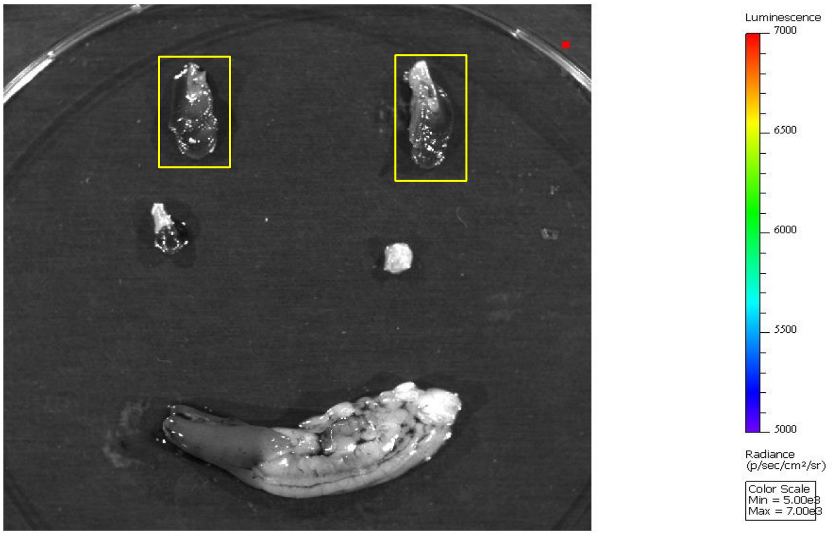

Brain

Intradermal
UK Luc ORF35 Del

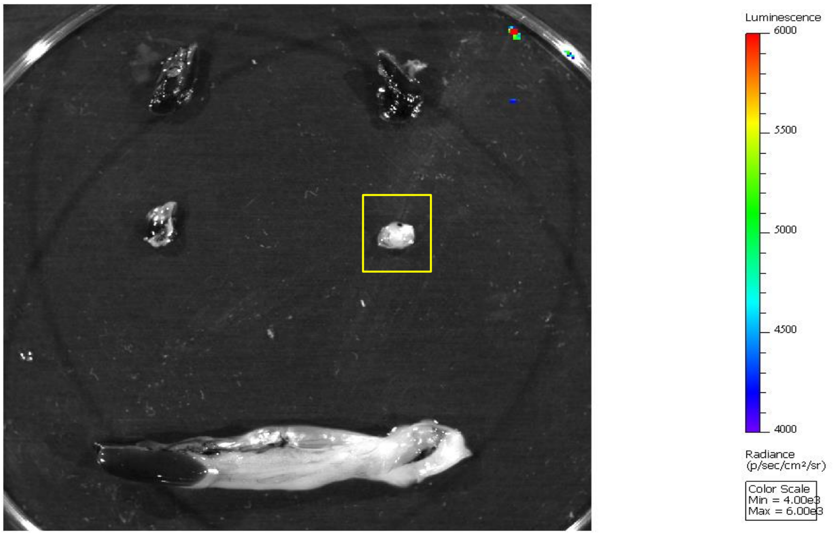

Figure 4C images

Eels with the closest scores to the mean of each group have been selected for illustration. The right end radiance scale was adjusted for each image. The images were cropped in the manuscript.

Skin left image

Mock

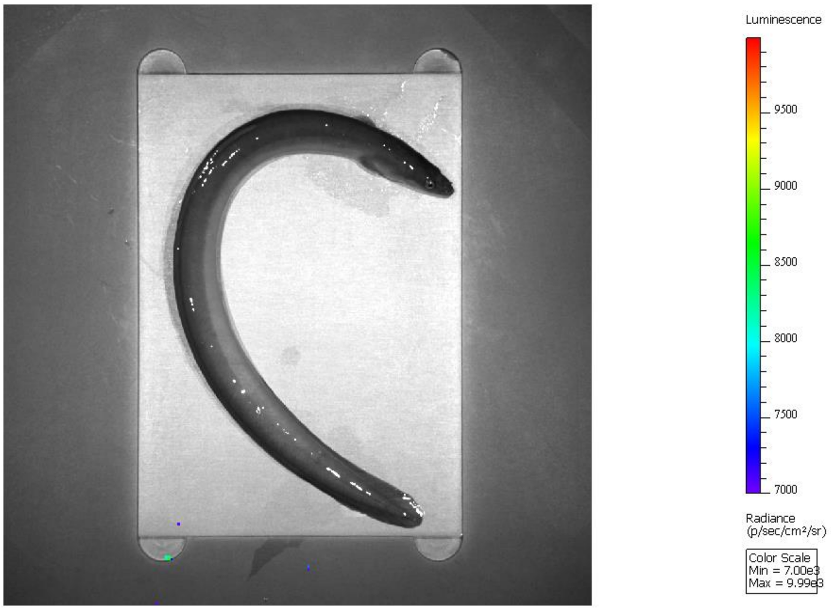

Skin right image

Mock

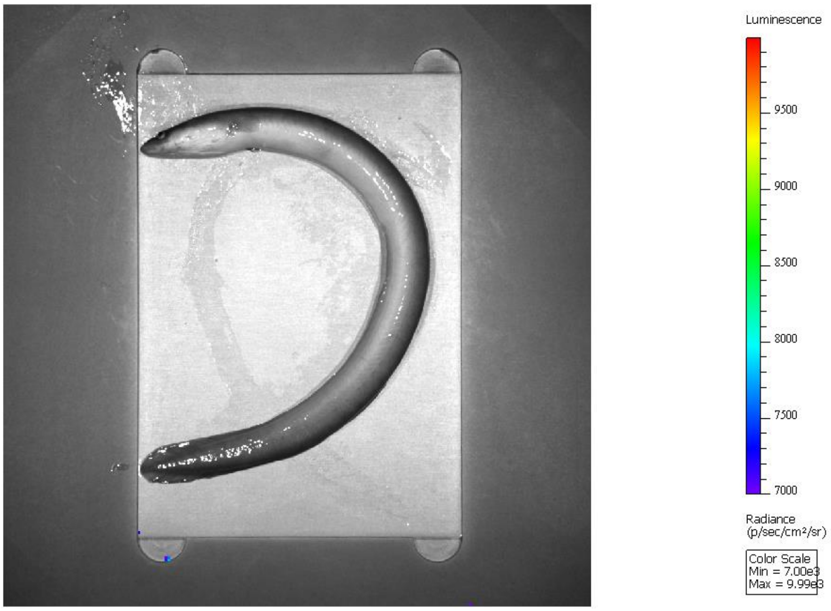

Figure 4C images

The yellow boxed gills and brain were taken for representing the group. The gills were rotated clockwise or anti-clockwise depending on the gills position to maintain consistency with the other group gills. The gills and brain images were cropped in the manuscript.

Gills

Mock

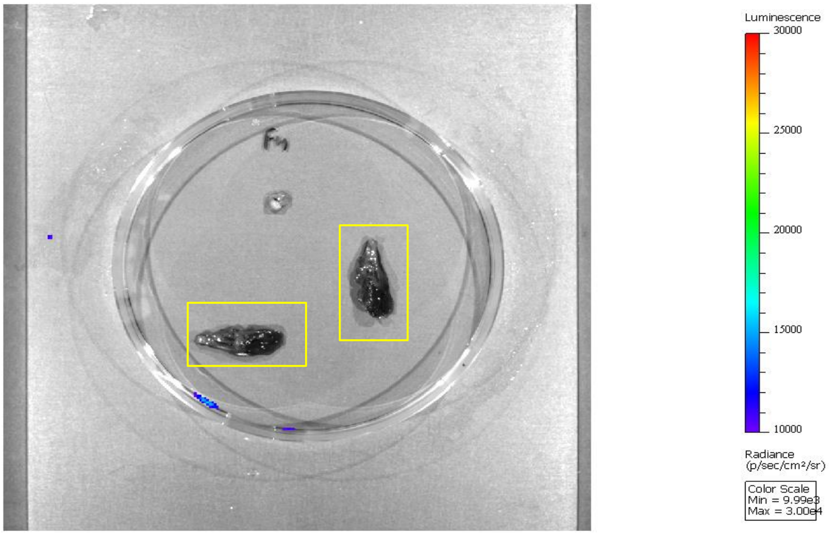

Brain

Mock

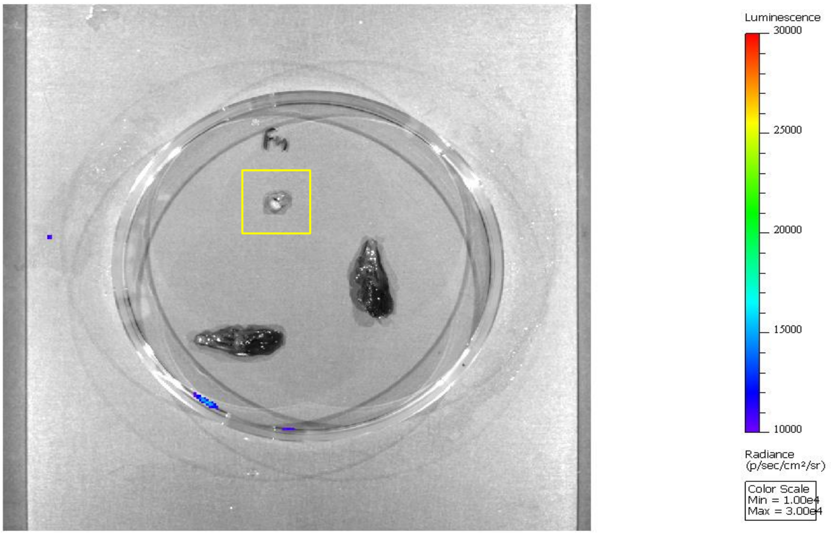

Figure 4C images

The images were cropped in the manuscript.

Skin left image

UK ORF35 Del
100,000 pfu/mL

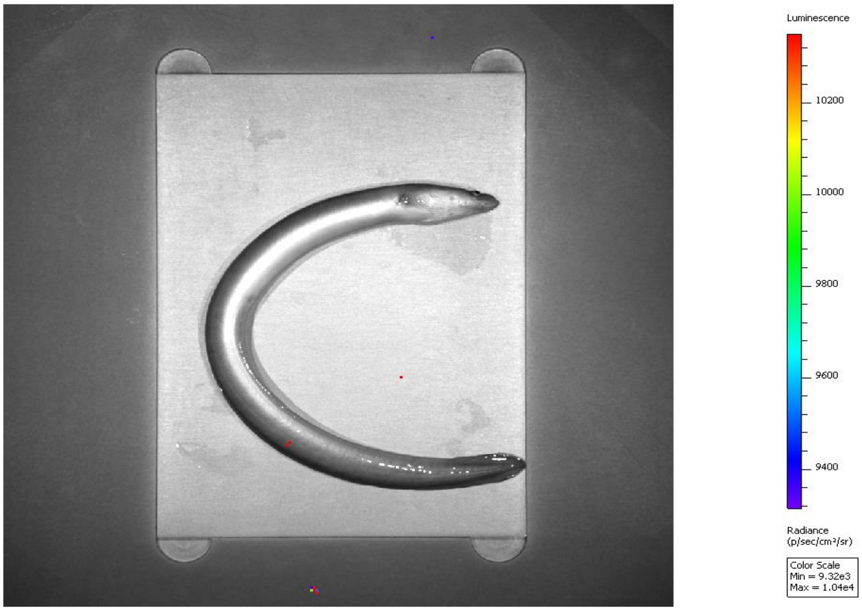

Skin right image

UK ORF35 Del
100,000 pfu/mL

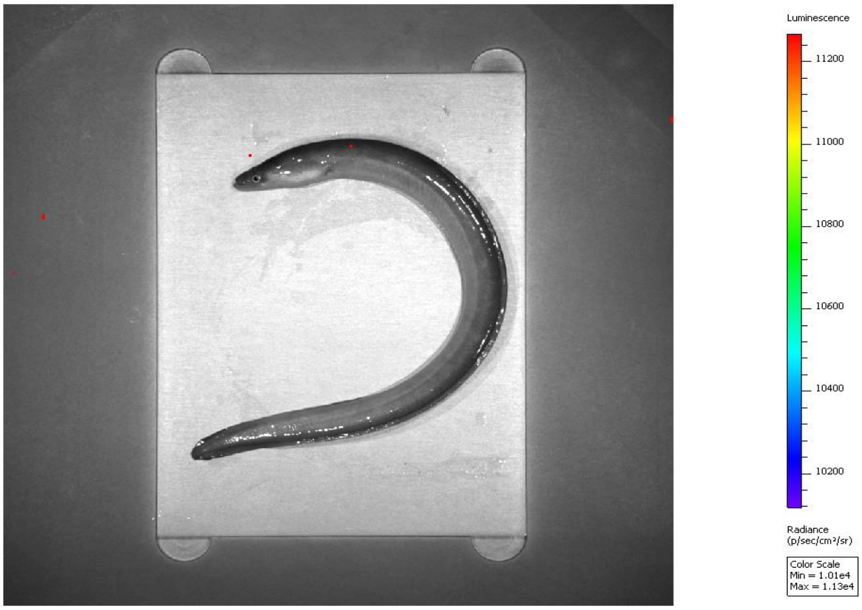

Figure 4C images

The yellow boxed gills, and brain were taken for representing the group. The right end radiance scale was adjusted. The images were cropped in the manuscript.

Gills

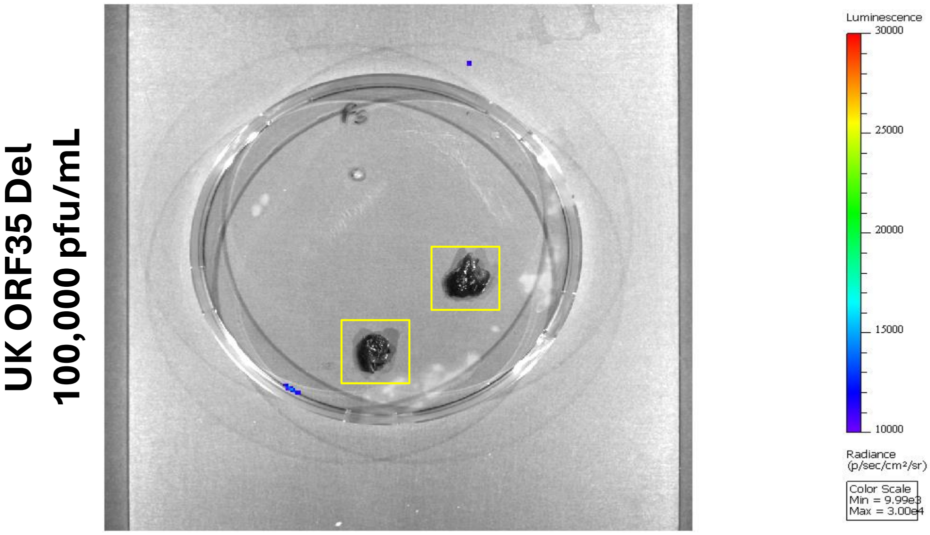

Brain

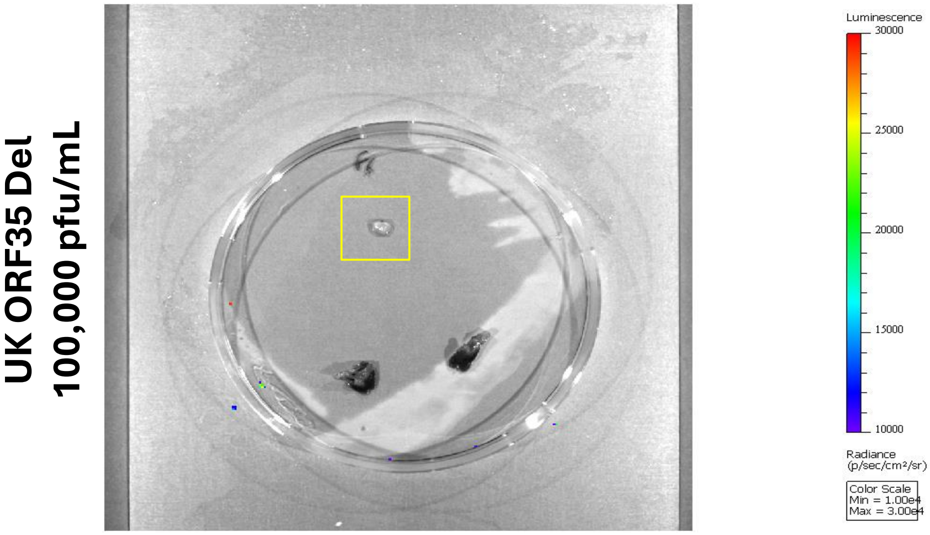

Figure 4C images

The images were cropped in the manuscript.

Skin left image

Psoralen/UV inactivated UK ORF35 Del 100,000 pfu/mL

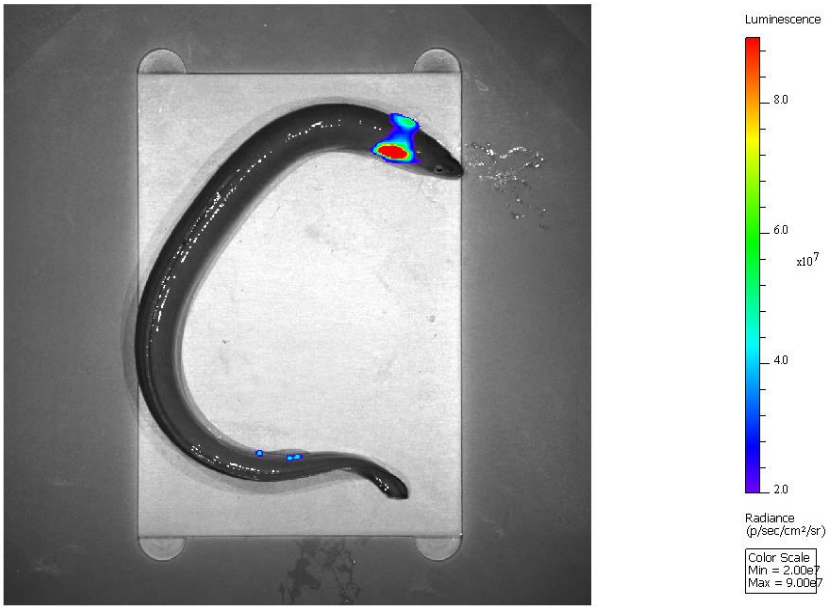

Skin right image

Psoralen/UV inactivated UK ORF35 Del 100,000 pfu/mL

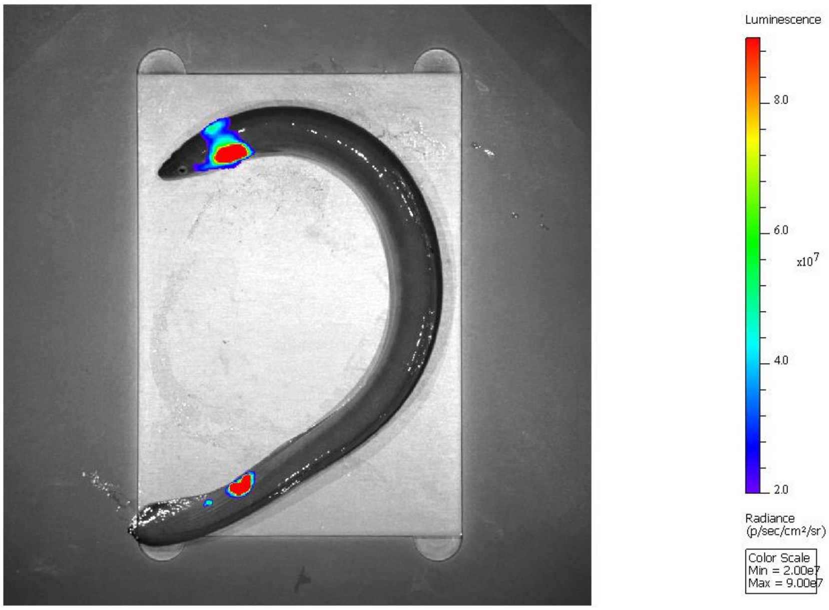

Figure 4C images

The yellow boxed gills, and brain were taken for representing the group. The right end radiance scale was adjusted. The images were cropped in the manuscript.

Gills

Psoralen/UV inactivated UK ORF35 Del 100,000 pfu/mL

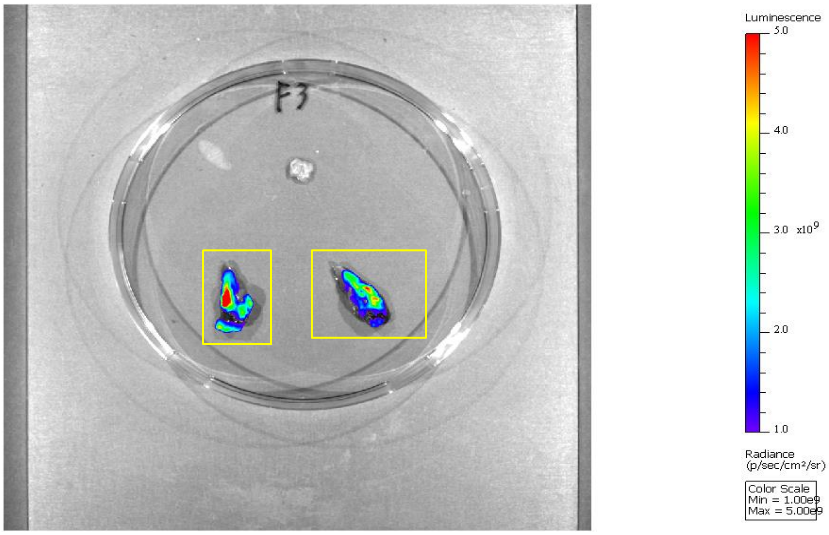

Brain

Psoralen/UV inactivated UK ORF35 Del 100,000 pfu/mL

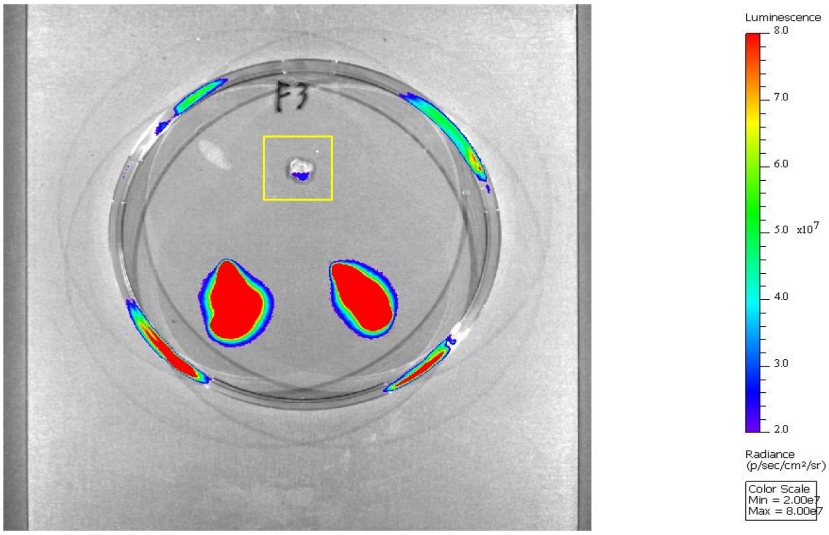

Figure 4C images

The images were cropped in the manuscript.

Skin left image

Positive Control

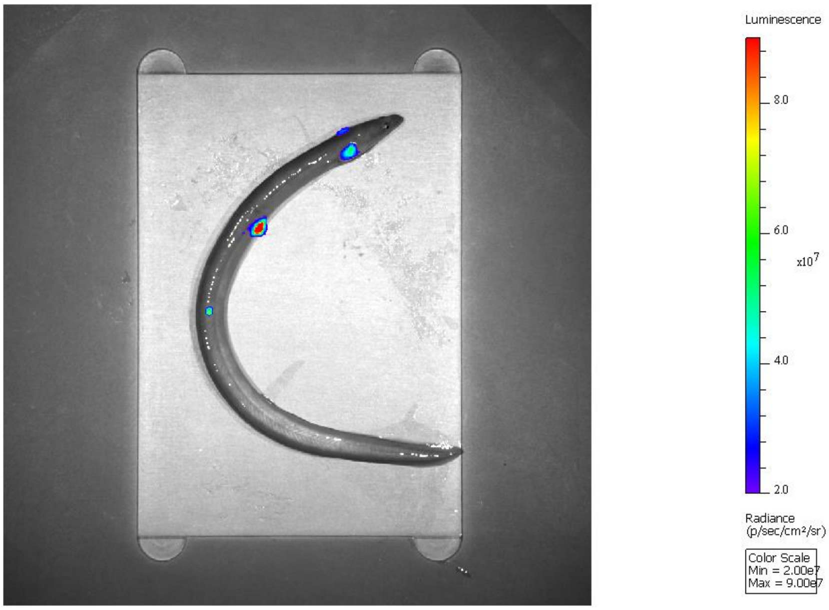

Skin right image

Positive Control

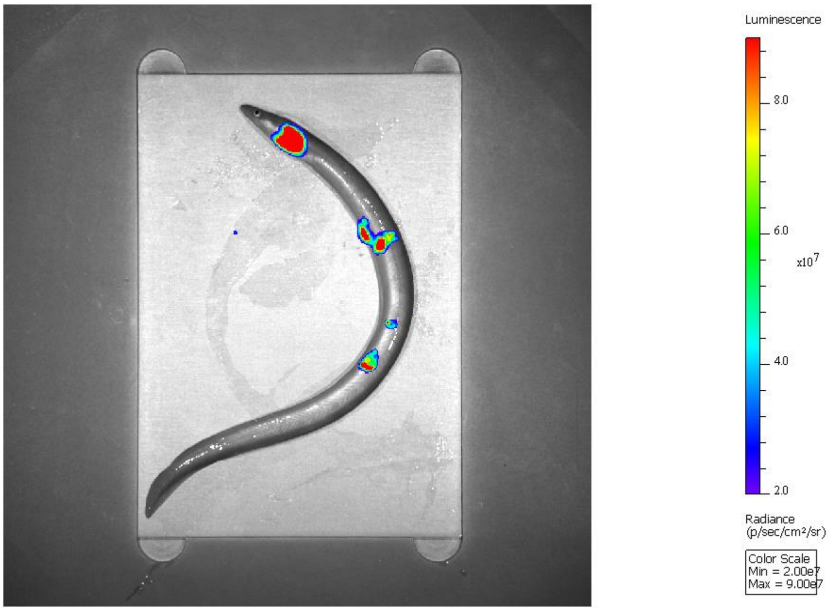

Figure 4C images

The yellow boxed gills, and brain were taken for representing the group. The right end radiance scale was adjusted. The images were cropped in the manuscript.

Gills

Positive Control

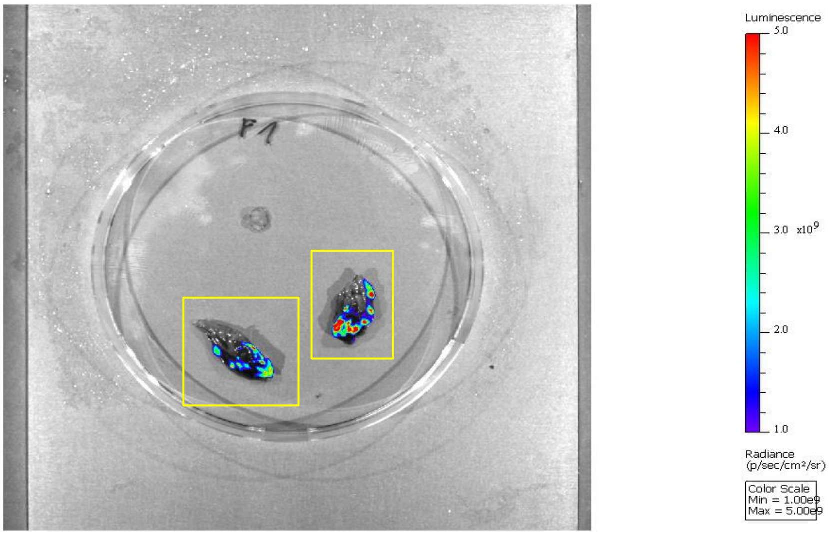

Brain

Positive Control

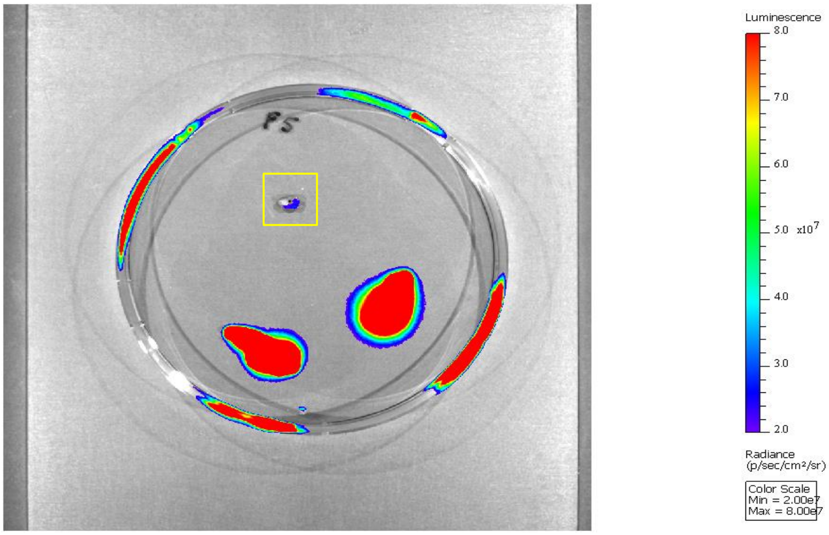

Supplement: Supplementary file 1 [file vaccines-12-01423-s001.zip › 20241209 Original images used in figures.pdf]
