# Supplementary material for: Development Using Bioluminescence Imaging of a Recombinant Anguillid Herpesvirus 1 Vaccine Candidate Associated with Normal Replication In Vitro but Abortive Infection In Vivo
Source: Vaccines (Basel). 2024 Dec 17;12(12):1423. doi: 10.3390/vaccines12121423 (PMC11728778; doi:10.3390/vaccines12121423)
Supplement: Supplementary file 1 [file vaccines-12-01423-s001.zip › 20241209 Table S1 FINAL.pdf]

Table S1. Oligonucleotide primers.

	Primer name	Sequence (5′- 3′)	Coordinates*
**Construction of ORF35 Del recombinant strains by homologous directed recombination**			
**Cassette name****			
pGEMT mCherry	Insert mCherry F	TTTTAGGACAATGGTGAGCAAGGGCGAG	
	Insert mCherry R	ACCACCACCATCACTTGTACAGCTCGTCC	
	H1-mCherry F	GGCGGCCGCGGGAATTCGATGGGCCAGGTTCTGGC CTC	
	H1-mCherry R	TGCTCACCATTGTCCTAAAAGTACCTGTCAAAAAA GCCTTTG	
	H2-mCherry F	GTACAAGTGATGGTGGTGGTGTGGTGGTG	
	H2-mCherry R	GCCGCGAATTCACTAGTGATGTACTTCTACTACTCT TTGCTCTTGGC	
**Transcription analysis**			
**Gene amplification**			
AngHV-1 ORF 32	AngHV1-ORF32 F	GCTCAAGCATCGATCAACAA	48648-48667
	AngHV1-ORF32 R	AGGCCGTTTCCAAAATCTCT	48962-48981
AngHV-1 ORF 33	AngHV1-ORF33 F	CACACACAACAGCAATGACG	49223-49242
	AngHV1-ORF33 R	CTCGCTTGCCACCTCTAATC	49581-49600
AngHV-1 ORF 34	AngHV1-ORF34 F	CTGACAGTGGGTTCCAAGC	53942-53960
	AngHV1-ORF34 R	ACGGTTTGCCTCAAAACCCT	54338-54357
AngHV-1 ORF 35	AngHV1-ORF35 F	ATCACGCCAAACTCATAGGG	54964-54983
	AngHV1-ORF35 R	CGTAGACGATGCGTTCCAAC	55244-55263
AngHV-1 ORF 36	AngHV1-ORF36 F	ACCGTTGTGTTTGTGTGC	56233-56250
	AngHV1-ORF36 R	CCACTTTGAAATCGTGAGGC	56643-56662
AngHV-1 DNA polymerase	HV DNAPol F	CATGCCGGGAGTCTTTTTGAT	88204-88224
	HV DNAPol R	GTGTCGGGCCTTTGTGGTGA	88578-88597

*Coordinates based on the reference AngHV-1 genome (GenBank accession number: MW580855.1).

**AngHV-1 sequences are underlined.

H1 and H2 in pGEMT mCherry: 500 bp sequences corresponding to the end of ORF34 and the ORF35-ORF36 intergenic region plus the end of ORF36, respectively.
